# Nigericin Boosts Anti-Tumor Immune Response via Inducing Pyroptosis in Triple-Negative Breast Cancer

**DOI:** 10.3390/cancers15123221

**Published:** 2023-06-16

**Authors:** Lisha Wu, Shoumin Bai, Jing Huang, Guohui Cui, Qingjian Li, Jingshu Wang, Xin Du, Wenkui Fu, Chuping Li, Wei Wei, Huan Lin, Man-Li Luo

**Affiliations:** 1 Department of Oncology, Sun Yat-sen Memorial Hospital, Sun Yat-sen University, Guangzhou 510120, China; 2 Guangdong Provincial Key Laboratory of Malignant Tumor Epigenetics and Gene Regulation, Guangdong-Hong Kong Joint Laboratory for RNA Medicine, Sun Yat-sen Memorial Hospital, Sun Yat-sen University, Guangzhou 510120, China; 3 Department of Breast and Thyroid Surgery, Peking University Shenzhen Hospital, Shenzhen 518036, China; 4 South China National Bio-Safety Laboratory, Zhongshan School of Medicine, Sun Yat-sen University, Guangzhou 510600, China; 5 Department of Breast Oncology, The Second Affiliated Hospital, Guangzhou University of Chinese Medicine, Guangzhou 510120, China; 6 Nanhai Translational Innovation Center of Precision Immunology, Sun Yat-sen Memorial Hospital, Foshan 528200, China

**Keywords:** nigericin, immune checkpoint inhibitor, pyroptosis, anti-tumor immune-response, triple-negative breast cancer

## Abstract

**Simple Summary:**

The response rate of advanced triple-negative breast cancer (TNBC) to immune checkpoint inhibitors remains unsatisfactory. Recent studies showed that inducing pyroptosis in tumor cells can amplify the anti-tumor immune response by turning “cold” tumors into “hot” tumors. Here, we demonstrated that the antibiotic nigericin caused TNBC cell death by inducing concurrent Caspase-1/GSDMD-mediated pyroptosis and Caspase-3-mediated apoptosis. Notably, we found that nigericin-induced pyroptosis promoted the infiltration and activation of T cells, as well as showing a synergistic therapeutic effect when combined with anti-PD-1 antibody treatment. This study provides a potential strategy to utilize nigericin to boost the anti-tumor immune responses required to treat advanced TNBC.

**Abstract:**

Although immune checkpoint inhibitors improved the clinical outcomes of advanced triple negative breast cancer (TBNC) patients, the response rate remains relatively low. Nigericin is an antibiotic derived from *Streptomyces hydrophobicus*. We found that nigericin caused cell death in TNBC cell lines MDA-MB-231 and 4T1 by inducing concurrent pyroptosis and apoptosis. As nigericin facilitated cellular potassium efflux, we discovered that it caused mitochondrial dysfunction, leading to mitochondrial ROS production, as well as activation of Caspase-1/GSDMD-mediated pyroptosis and Caspase-3-mediated apoptosis in TNBC cells. Notably, nigericin-induced pyroptosis could amplify the anti-tumor immune response by enhancing the infiltration and anti-tumor effect of CD4+ and CD8+ T cells. Moreover, nigericin showed a synergistic therapeutic effect when combined with anti-PD-1 antibody in TNBC treatment. Our study reveals that nigericin may be a promising anti-tumor agent, especially in combination with immune checkpoint inhibitors for advanced TNBC treatment.

## 1. Introduction

Triple-negative breast cancer (TNBC) represents the most malignant and aggressive subtype of breast cancer, which lacks expression of estrogen receptor, progesterone receptor and Her-2 [1,2]. Due to a lack of therapeutic targets and frequent recurrence or progression after chemotherapy, TNBC is considered the most challenging breast cancer subtype to treat [3,4]. Compared to other breast cancer subtypes, TNBC has a higher mutation burden, more immune cells infiltration and higher programmed cell death ligand 1 (PD-L1) expression [5]. Although Impassion130 and TONIC clinical trials demonstrated that patients could benefit from immune checkpoint inhibitors, the response rates of anti-programmed cell death 1 (PD-1)/PD-L1 antibodies alone or combined with chemotherapy in advanced TNBC patients remain unsatisfactory [6,7]. Thus, there is an urgent demand for developing a new strategy to improve the therapeutic effect of checkpoint inhibitors for TNBC treatment.

Mitochondria play essential roles in cellular metabolism. Multiple metabolites of mitochondria were reported to contribute to tumor progression and adaptation to treatment [8,9]. Mitochondria are the major source of reactive oxygen species (ROS), which are the by-product of oxygen consumption and cellular metabolism. The mismanagement of ROS, which is associated with mitochondrial dysfunction and oxidative stress, can cause defects in mtDNA repair system and mitochondrial nucleoid protection, which is, in turn, linked to tumorigenesis in breast cancer [8,10].

Pyroptosis refers to a programmed necrosis featuring membrane pore-forming and blebbing, cell swelling and eventual cell lysis [11]. In contrast to apoptosis, pyroptosis is accompanied by the release of large numbers of pro-inflammatory factors, such as interleukin (IL)-1β and IL-18, thus inducing a strong immune response [12,13]. A previous study showed that inducing pyroptosis in only 10–20% of tumor cells in cancer tissues is sufficient to eliminate almost all tumor cells [14], suggesting that pyroptosis can amplify the anti-tumor immune response by turning “cold” tumors into “hot” tumors [15,16]. Thus, inducing pyroptosis is a potential strategy to boost the anti-tumor immune response through combination with immune checkpoint inhibitors.

Nigericin is an antibiotic derived from *Streptomyces hygroscopicus* that acts as an ionophore, which causes efflux of potassium from the cell and, thus, influences the mitochondria membrane potential [17,18]. Recent studies revealed that nigericin can selectively target cancer stem cells of breast, nasopharyngeal, lung and colorectal cancers [19,20,21]. Inactivation of Wnt/β-catenin pathway was found to be one of the anti-tumor mechanisms of nigericin [22]. In addition to the above anti-tumor mechanisms, nigericin was demonstrated to induce pyroptosis in macrophages by activating NLRP3 inflammasome [23] and Caspase-1 cleavage [24]. In this study, we found that nigericin could induce TNBC cell death, and we further investigated the underlying mechanism, as well as the synergistic anti-tumor effect of nigericin with the PD-1 antibody treatment.

## 2. Materials and Methods

### 2.1. Cell Culture and Reagents

Human breast cancer cell MDA-MB-231, MDA-MB-468, SK-BR-3 and MCF-7, T47D and mouse breast cancer cell 4T1 were obtained from the American Type Culture Collection (ATCC). MDA-MB-231, MDA-MB-468, SK-BR-3, MCF-7 and 4T1 were cultured in DMEM supplemented with 10% FBS. T47D was cultured in RPMI-1640 supplemented with 10% FBS. Nigericin (Sigma, St. Loius, MI, USA, Cat#28643-80-3) was prepared as a 5 mg/mL (6.7 mM) stock solution in 100% ethanol and diluted by adding an appropriate amount of endotoxin-free saline or culture medium. The ultimate working concentrations of nigericin were 0, 0.25, 0.5, 1, 2, 5, 10 and 20 μg/mL. Small-molecule inhibitors, including necrostatin-1 (necroptosis inhibitor, Cat#S8037), ferrostatin-1 (ferroptosis inhibitor, Cat#S7243), chloroquine (autophagy inhibitor, Cat#S6999) and z-VAD (pan-Caspase inhibitor, Cat#S7023) were purchased from Selleckchem. The working concentrations of each inhibitor were 10 μM, 20 μM, 20 μM and 50 μM, respectively.

### 2.2. Lactate Dehydrogenase (LDH) Release Assay and IL-1β ELISA Assay

For LDH detection, cells were seeded in 96-well culture plates at a density of 5 × 10^4^ cells/well and treated with nigericin (2 μg/mL) for 0 h, 12 h, 24 h and 48 h. LDH levels in the supernatant were measured using a CytoTox96 LDH-release kit (Promega, Madison, WI, USA, Cat#PR-G1780). The percentage of LDH release was calculated using the equation (LDHsample − LDHbackground)/(LDHmaximum − LDHbackground) × 100%. The absorbance value at 490 nm was then measured. For IL-1β detection, cells were seeded in 96-well culture plates at a density of 5 × 10^4^ cells/well and treated with nigericin (2 μg/mL) for 24 h. IL-1β level was detected using the IL-1β ELISA kit (Invitrogen, Waltham, MA, USA, Cat#BMS224-2 or BMS6002) according to the manufacturer’s instructions. The absorbance value at 620 nm was then measured.

### 2.3. Western Blot

Cells were harvested and lysed in RIPA buffer (Cwbiotech, Beijing, China, Cat#CW2333S) supplemented with proteinase (Cwbiotech, Beijing, China, Cat#CW2200S) and phosphatase inhibitors (Cwbiotech, Beijing, China, Cat#CW2383S). The lysates (20 μg protein) were boiled with sample buffer, separated via SDS-PAGE and transferred to PVDF membrane. All membranes were blocked with 5% nonfat milk in PBS with 0.1% Tween-20 (PBS-T) for 1 h and incubated at 4 °C overnight with the primary antibodies. After being washed with PBS-T, membranes were incubated with HRP-conjugated secondary antibodies and analyzed via chemiluminescence. The antibodies and dilution ratios were listed in Table S1.

### 2.4. Scanning Electron Microscopy (SEM)

Cells were rinsed with PBS twice and fixed with 2.5% glutaraldehyde overnight. Sample were dehydrated through a graded series of ethanol (30%, 50%, 70%, 95% and 100%) and dried via the tertiary butanol method. Dried specimens were sputter coated with gold-palladium and imaged with a JEOL JSM-6390LV field emission scanning electron microscope operating at 10 kV.

### 2.5. Immunofluorescence Microscopy

Cells grown on confocal dishes were fixed with 4% paraformaldehyde for 20 min, followed by permeabilization for 20 min in 0.1% Triton X-100 and blocking using 5% BSA for 1 h. Next, the cells were stained with the diluted primary antibody anti-GSDMD N-terminal, as listed in Table S1, followed by incubation with the secondary antibody Alexa Fluor^®^ 594 Conjugate-labeled anti-rabbit IgG (Cell Signaling Technology, Danvers, MA, USA) at room temperature for 1 h. Nuclei were counterstained with DAPI (Cell Signaling Technology, Danvers, MA, USA). Images were captured using a confocal microscope system (Zeiss LSM 780, Jena, Germany).

### 2.6. siRNA Knockdown

Cells were seeded in six-well plates to be 70% confluent, before being transfected with siRNAs (Ige Biotechnology Ltd., Guangzhou, China) targeting Caspase-1, Caspase-3, GSDMD, GSDME or negative control siRNA using Lipofectamine 3000 according to the manufacturer’s instructions. After 48 h transfection, MDA-MB-231 and 4T1 cells were subjected to subsequent analyses. The sequence of siRNA was listed in Table S2.

### 2.7. Detection of Mitochondrial Membrane Potential Changes

Mitochondrial membrane potential was detected using a 5,5′,6,6′-tetrachloro-1,1′,3,3′-tetraethylbenzimidazolyl-carbocyanine iodide (JC-1) probe kit (Thermo Fisher, Waltham, MA, USA, Cat#M34152) according to manufacturer’s instructions. The JC-1 probe changed its fluorescent properties based on the mitochondrial membrane potential. At high mitochondrial membrane potential, JC-1 aggregated and yielded red-colored emission (590 nm). At low mitochondrial membrane potential, JC-1 was predominantly a monomer that yielded green-colored emission (530 nm).

### 2.8. Detection of Cellular ROS

The levels of cellular ROS were determined using the Reactive Oxygen Species Assay Kit (Beyotime, Nantong, China, Cat#S0033S) according to the manufacturer’s instructions. Intracellular ROS levels were determined via probe 2′,7′-dichlorodihydrofluorescein diacetates (DCFH-DA) with flow cytometry. DCFH-DA was oxidized to dihydrodichlorofluorescein (DCF) via cellular ROS. TNBC cells were treated in indicated conditions, followed by incubation with DCFH-DA at 37 °C for 20 min. Next, cells were collected, and we analyzed the green fluorescence of DCF with a FACSCalibur flow cytometer.

### 2.9. Animal Experiments

Six-to-eight-week old female immunocompetent BALB/c mice were purchased from Guangdong Medical Laboratory Animal Center (Guangzhou, China). All mice were maintained in Sun Yat-en University’s (SYSU) animal facilities, as approved by the Institutional Animal Care and Use Committee (IACUC) of SYSU (SYSU-IACUC-2022-B0964). Next, 5 × 10^5^ 4T1 cells were injected orthotopically into the left inguinal mammary fat pad of BALB/c mice. Mice were randomly divided into four groups and treated at the same time with indicated drugs. Nigericin (2 mg/kg) was injected subcutaneously every two days, and anti-PD-1 (BioXcell, Lebanon, NH, USA, Cat#BE0033-2, 250 μg/mouse) was injected intraperitoneally every week. Tumor volumes were monitored every three-to-four days. At the end of the experiment (after about 4 weeks), the mice were sacrificed via carbon dioxide euthanasia, and tumors were harvested for further analysis.

### 2.10. Immuno-Histochemistry (IHC)

Next, 4% paraformaldehyde (PFA) was used to fix tumor tissues. After being embedded in paraffin, tissue specimens were cut into 4-μm sections. Sections were deparaffinized with xylene, rehydrated in graded ethanol and incubated in sodium citrate buffer (10 mM, pH 6.0 at 95 °C 60 min) for antigen retrieval. The sections were blocked with 5% goat serum, before being incubated overnight at 4 °C with primary antibodies (listed in Table S1). The SP-9000 Detection Kits (ZSGB-BIO, China, Cat#SP-9000) and DAB Kit (ZSGB-Bio, Beijing, China, Cat#ZLI-9019) were used to stain the sections.

### 2.11. Mononuclear Cells Suspension Preparation and Flow Cytometry Staining

The single-cell suspension from tumor tissues was prepared as previously described [25]. FITC-, PE-, APC- or PerCP-labeled antibodies to CD45, CD4, CD8, TNF-α were listed in Table S1. Cells were, firstly, stimulated with 5 ng/mL phorbol 12-myristate 13-acetate (PMA) and 500 ng/mL ionomycin at 37 °C for 2 h, followed by 1 µL Monensin for 2 h. Next, the cells were stained for surface markers with antibodies in phosphate-buffered saline (PBS) with 2% fetal calf serum (FCS) on ice for 30 min. After washing with PBS, cells were either analyzed using a FACSCalibur flow cytometer or further fixed and stained with cytokine antibodies. Cells were resuspended and incubated with 200 µL Cytofix/Cytoperm solution (BD Biosciences, Franklin Lakes, NJ, USA, Cat#554722) at 4 °C for 20 min, before being washed in permeabilization buffer (BD Biosciences, Cat#554723) twice and stained with antibodies against cytokines. Data were analyzed using FlowJo software Version 10.

### 2.12. Statistical Analysis

Statistical analyses were performed with GraphPad Prism 8.3.0. Data are presented as mean ± SD. Student’s *t*-test, one-way ANOVA or two-way ANOVA were performed to compare the differences between the groups. Two-sided *p*-values were calculated, and *p* < 0.05 was considered statistically significant. In all cases, * *p* < 0.05, ** *p* < 0.01, *** *p* < 0.001, ns, not significant.

## 3. Results

### 3.1. Nigericin Induces Concurrent Apoptotic and Pyroptotic Cell Death in TNBC Cells

**Figure 1:**
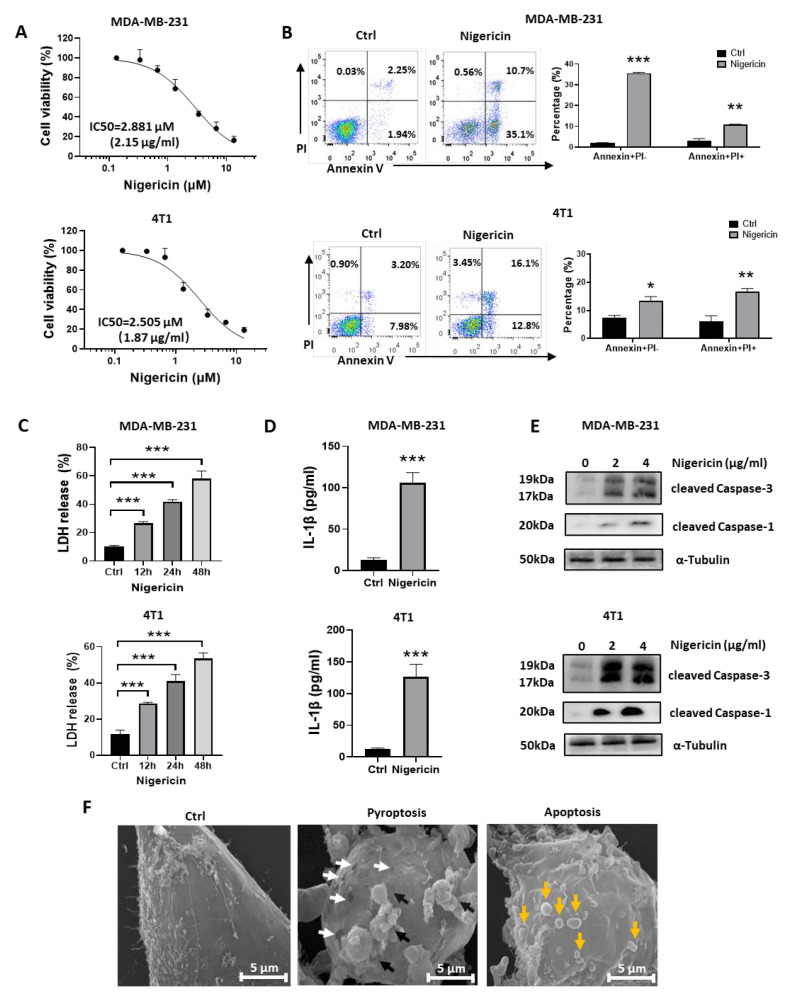
Nigericin-treated TNBC cells exhibit both pyroptotic and apoptotic features. (**A**) Human TNBC cell line MDA-MB-231 and mouse TNBC cell line 4T1 were exposed to increasing concentrations of nigericin (0, 0.25, 0.5, 1, 2, 5, 10 and 20 μg/mL) for 24 h. Cell viability was measured using MTS assays, and IC50 value was calculated based on cell viability curve. Displayed are mean ± SD relative to control. (**B**) Flow cytometry analysis of PI and Annexin V stained TNBC cells before and after nigericin treatment (nigericin 2 μg/mL for 12 h, Annexin V+PI−: early apoptotic cells, Annexin V+PI+: late apoptotic/necrotic cells). Right panel shows cell percentages in left flow cytometry plots. Data are shown as mean ± SD (*t*-test, * *p* < 0.05, ** *p* < 0.01, *** *p* < 0.001). (**C**) LDH released from TNBC cells upon nigericin treatment (2 μg/mL) at different time points was detected using the LDH release assay. Each bar represents mean ± SD of experimental triplicates (one-way ANOVA, *** *p* < 0.001). (**D**) IL-1β released from TNBC cells upon nigericin treatment (2 μg/mL for 24 h) was detected with the IL-1β ELISA. Each bar represents mean ± SD of experimental triplicates (*t*-test, *** *p* < 0.001). Level of cleaved Caspase-1 or cleaved Caspase-3 was detected via western blots. (**E**) Nigericin treatment induced the cleavage of Caspase-1 and Caspase-3, as detected by western blots. (**F**) Representative SEM pictures of MDA-MB-231 treated with nigericin (2 μg/mL for 24 h). White arrows indicate membrane pores. Black arrows indicate pyroptotic bubbles. Yellow arrows indicate apoptotic bodies. TNBC, triple-negative breast cancer; PI, propidium iodide; LDH, lactate dehydrogenase; IL, interleukin; SEM, scanning electron microscope.

Nigericin suppressed the cell viability of MDA-MB-231 and 4T1 cells in a dose-dependent manner, recording IC50 values of 2.881 μM and 2.505 μM, respectively (Figure 1A); nigericin also inhibited the colony formation of these cells (Figure S1A). Flow cytometric analysis indicated that nigericin increased early apoptosis (Annexin V+PI−) and late apoptosis/necrosis (Annexin V+PI+) in TNBC cells (Figure 1B). After nigericin treatment, some TNBC cells showed apoptosis morphology, such as cell shrinkage, while some of the other cells showed swelling, which was a characteristic of pyroptosis (Figure S1B). The release of lactate dehydrogenase (LDH), which was caused by cell membrane rupture, is a hallmark of necrotic cell death, including pyroptosis [26]. Pyroptosis also leads to the secretion of inflammatory factors, such as IL-1β [12,13]. We found that the levels of LDH and IL-1β in supernatant increased upon nigericin treatment (Figure 1C,D). Together, these results suggested that nigericin-treated TNBC cells exhibited both pyroptotic and apoptotic features.

Caspase family plays a crucial role in inducing apoptotic or pyroptotic death. Caspase-1 induces gasdermin-D (GSDMD)-mediated pyroptosis [11,12], and Caspase-3 is known as the executor of apoptosis [27]. Recently, Caspase-3 was also reported to trigger pyroptosis through gasdermin-E (GSDME) [28]. In this study, we observed that nigericin could induce both Caspase-1 and Caspase-3 cleavage (Figure 1E). Consistently, we found that nigericin-induced death in TNBC cells could be prevented using z-VAD (pan-Caspase inhibitor), but not by necrostatin-1 (necroptosis inhibitor), ferrostatin-1 (ferroptosis inhibitor) or chloroquine (autophagy inhibitor) (Figure S1B), which further verified that nigericin-induced pyroptotic and apoptotic death in TNBC cells occurred in a Caspase-dependent manner. With scanning electron microscope (SEM), we also observed the pyroptotic (pore-forming membranes and bubbles) and apoptotic features (apoptotic bodies) in TNBC cells upon nigericin treatment (Figure 1F). Together, these results indicated that nigericin induced concurrent pyroptosis and apoptosis in TNBC cells.

### 3.2. Nigericin Induces TNBC Cell Pyroptosis via Caspase-1/GSDMD-Dependent Pathway

**Figure 2:**
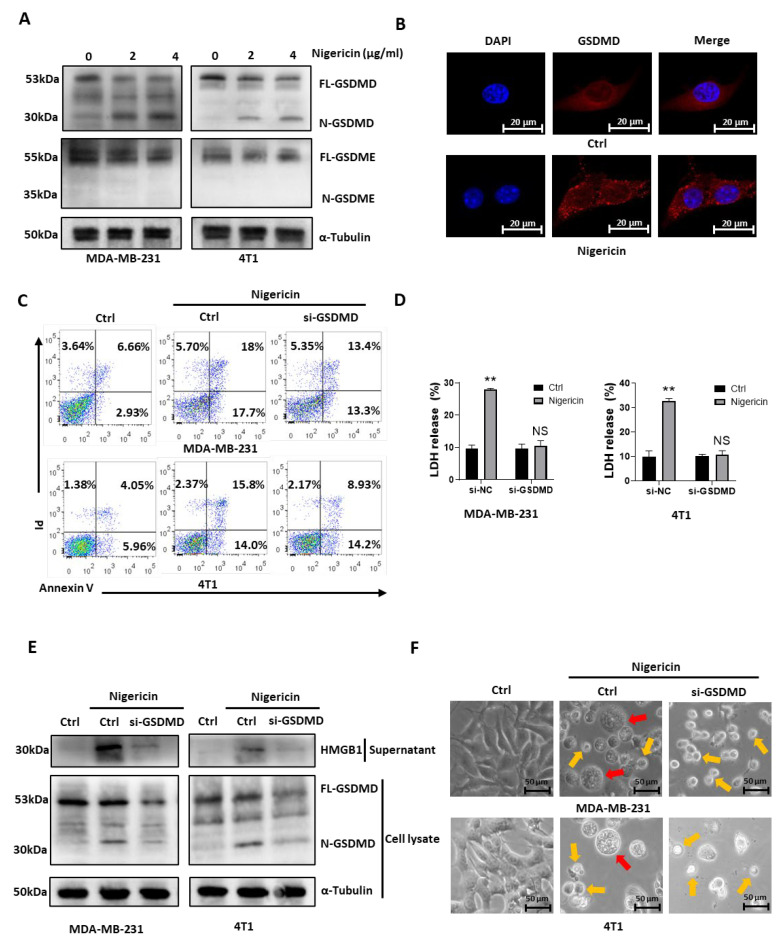
Nigericin-induced TNBC pyroptosis is GSDMD-dependent. (**A**) Western blots detected GSDMD or GSDME in TNBC cells upon nigericin treatment (2 μg/mL or 4 μg/mL for 12 h). (**B**) Confocal microscopy detected the N-terminal GSDMD migration from cytoplasm to membrane in MDA-MB-231 cells upon nigericin treatment (2 μg/mL for 12 h). (**C**) Flow cytometry detected PI- and Annexin V-stained TNBC cells with and without GSDMD knockdown, which occurred upon nigericin treatment (nigericin 2 μg/mL for 12 h, Annexin V+PI−: early apoptotic cells; Annexin V+PI+: late apoptotic/necrotic cells). (**D**) LDH released from TNBC cells with or without GSDMD knockdown, treated with nigericin (2 μg/mL for 12 h). Bar graphs represent mean ± SD of experimental triplicates (*t*-test, ** *p* < 0.01). (**E**) Western blots detected supernatant HMGB1 and cytoplasm GSDMD in control and GSDMD knockdown TNBC cells treated with nigericin (2 μg/mL for 12 h). (**F**) Representative phase-contrast images of control and GSDMD knockdown TNBC cells treated with nigericin (2 μg/mL for 24 h). Red arrows indicate pyroptotic cells, and yellow arrows indicate apoptotic cells. TNBC, triple-negative breast cancer; FL-GSDMD, full-length gasdermin-D; N-GSDMD, N-terminal fragments of gasdermin-D; FL-GSDME, full-length gasdermin-E; N-GSDME, N-terminal fragments of gasdermin-E; DAPI, 4′,6-diamidino-2-phenylindole; PI, propidium iodide; LDH, lactate dehydrogenase; HMGB1, high mobility group box-1.

We then investigated the underlying mechanism of nigericin-induced TNBC cell pyroptosis. As both cleaved Caspase-1 and cleaved Caspase-3 were elevated with nigericin treatment (Figure 1E), we detected their downstream factors GSDMD and GSDME [12,28]. Western blots showed that nigericin led to the cleavage of GSDMD but not of GSDME. The gathering of N-terminal fragments of gasdermin on the cellular membrane is the key step required for gasdermin to activate pyroptosis [11,12,13]. The level of N-GSDMD increased after nigericin treatment (Figure 2A). Next, using immunofluorescence confocal microscopy, we observed that N-terminal fragments of GSDMD migrated from cytoplasm to membrane in nigericin-treated cells (Figure 2B). These results suggested that nigericin induced pyroptosis through the Caspase-1/GSDMD pathway. Consistent with these results, the nigericin-induced necrosis (Figure 2C) and the release of LDH (Figure 2D) were decreased by knocking down GSDMD. In addition, high mobility group box-1 (HMGB1), i.e., the ubiquitous nuclear protein released by necrotic cells that served as an indicator of pyroptosis [29], also decreased in the supernatant of GSDMD knockdown cells (Figure 2E). Moreover, nigericin treated TNBC cells did not show pyroptotic morphology with GSDMD knocking down (Figure 2F). Indeed, MDA-MB-231 expressed a moderate level of GSDMD, and 4T1 cells expressed a high level of GSDMD (Figure S2A). The cell line SK-BR-3 expressing low level of GSDMD did not exhibit pyroptotic features after nigericin treatment (Figure S2B). As for the TNBC cell line MDA-MB-468, with a moderate level of GSDMD expression, similar morphologic changes and cleaved N-terminal fragments of GSDMD were observed upon nigericin treatment (Figure S2C,D). Although we did not observe the nigericin-induced cleavage of N-terminal fragments of GSDME, we still knocked down GSDME to see whether it would abolish nigericin-induced pyroptosis. We found nigericin-induced morphological changes and the release of LDH were not reversed in GSDME knockdown cells (Figure S2E,F). Therefore, GSDMD, but not GSDME, was responsible for nigericin induced-pyroptosis in TNBC cells.

### 3.3. Nigericin Causes Mitochondrial Dysfunction in TNBC Cells

**Figure 3:**
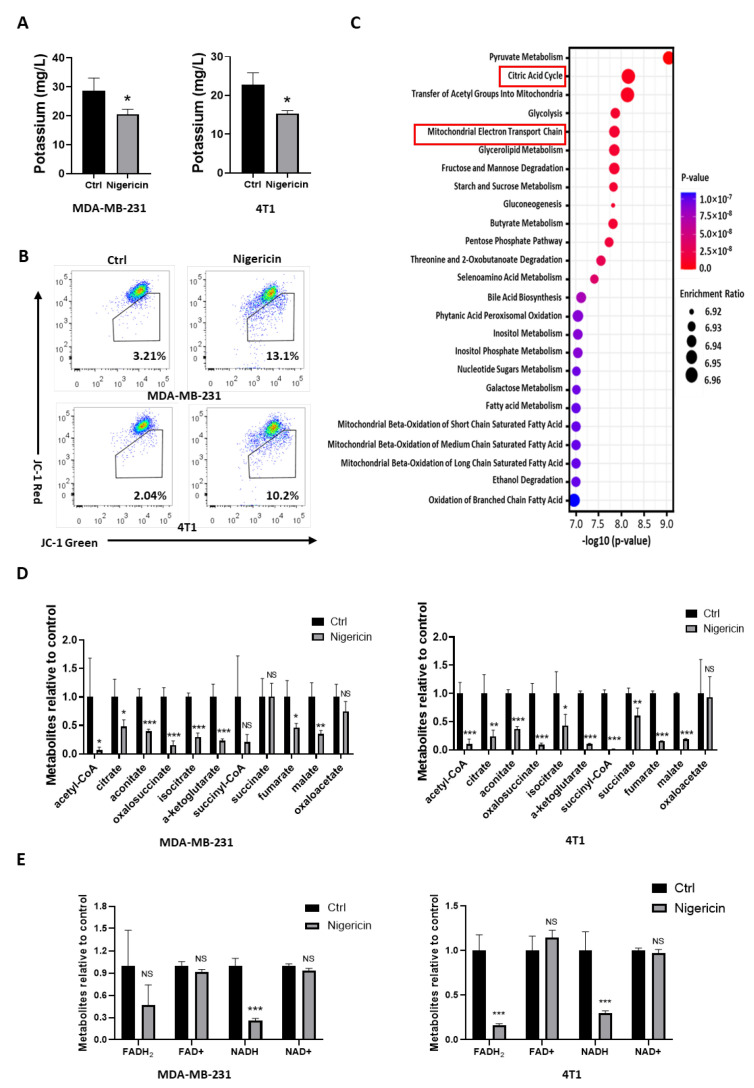
Nigericin treatment leads to mitochondrial dysfunction. (**A**) Changes in intracellular potassium level in TNBC cells before and after nigericin treatment (2 μg/mL for 6 h), as detected via ICP-MS. Bar graphs stand for mean ± SD of experimental triplicates (*t*-test, * *p* < 0.05). (**B**) Mitochondrial membrane potential changes in TNBC cells (nigericin 2 μg/mL for 6 h), as detected via JC-1 probes. At high mitochondrial membrane potential, JC-1 aggregated and yielded red-colored emission (590 nm). At low mitochondrial membrane potential, JC-1 was predominantly a monomer that yielded green-colored emission (530 nm). (**C**) Metabolites screened via mass spectrometry in 4T1 cells with or without nigericin treatment. Metabolic pathway enrichment of differential metabolites was performed based on Metaboanalyst (https://www.metaboanalyst.ca (accessed on 31 October 2020)). (**D**,**E**) Nigericin significantly impacted mitochondrial metabolism. (**D**) Metabolites of TCA cycle and (**E**) oxidative phosphorylation upon nigericin treatment detected via mass spectrometry. Bar graphs stand for mean ± SD of experimental quadruplicates (*t*-test, * *p* < 0.05, ** *p* < 0.01, *** *p* < 0.001). TNBC, triple-negative breast cancer; JC-1, tetraethylbenzimidazolylcarbocyanine iodide; ICP-MS, inductively coupled plasma mass spectrometry; TCA cycle, tricarboxylic acid cycle; FADH2, flavin adenine dinucleotide; NADH, nicotinamide adenine dinucleotide.

We then sought to elucidate the mechanism of how nigericin induced pyroptosis in TNBC cells. Nigericin works as an ionophore, which causes efflux of potassium from the cell [17,18]. Using inductively coupled plasma mass spectrometry (ICP-MS), nigericin was shown to reduce intracellular potassium in both MDA-MB-231 and 4T1 cells (Figure 3A). Specific to mitochondria, nigericin-induced K+ efflux was accompanied by H+ uptake, which uncoupled oxidative phosphorylation and inhibited the mitochondria respiration by decreasing the membrane potential [30,31]. JC-1 probes are widely used to detect the mitochondrial membrane potential change [32]. After nigericin treatment, the proportion of JC-1-labeled TNBC cells in green fluorescence increased, suggesting that the mitochondrial membrane potential decreased (Figure 3B). We then screened the metabolites associated with mitochondrial metabolism via mass spectrometry, finding that the metabolites in tricarboxylic acid (TCA) cycle were the most significantly affected of all metabolic pathways in nigericin-treated TNBC cells (Figure 3C). All metabolites in the TCA cycle decreased (Figure 3D). Consistent with the above results, FADH2 and NADH also decreased upon nigericin treatment (Figure 3E), supporting the idea that oxidative phosphorylation was impaired after nigericin treatment. These results indicated that nigericin treatment induced the mitochondrial dysfunction.

### 3.4. Nigericin-Mediated Mitochondria Dysfunction Induces Caspase-1 Activation and Pyroptosis

**Figure 4:**
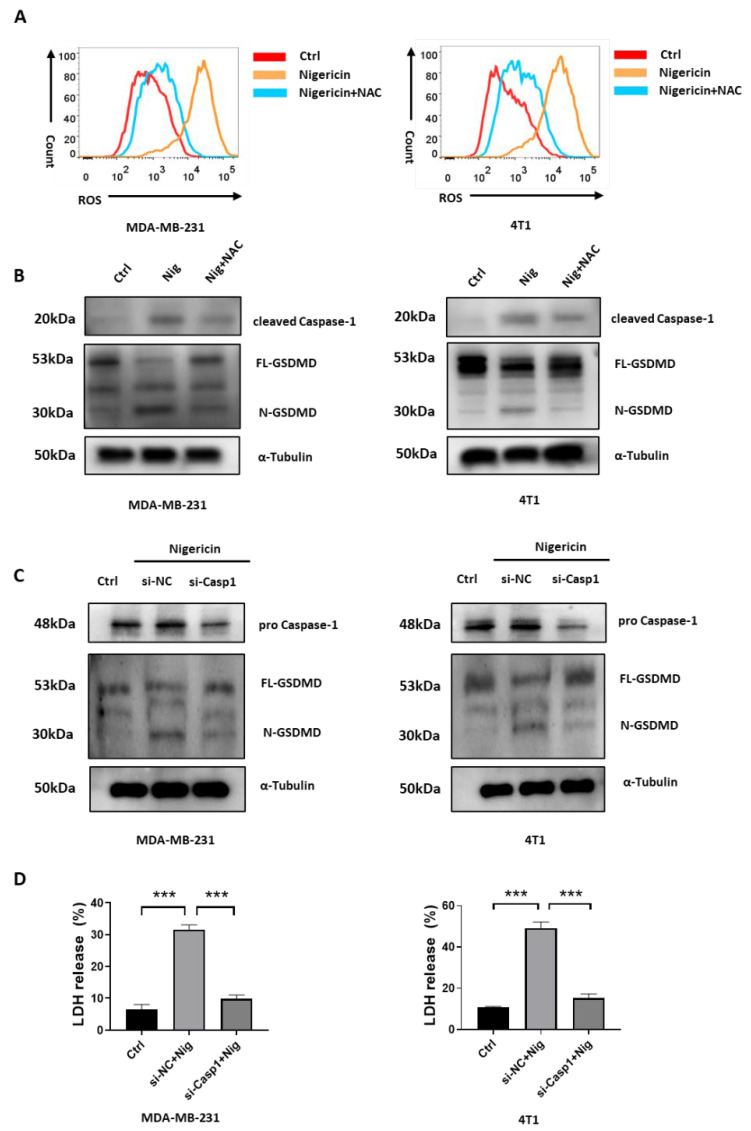
Nigericin-mediated mitochondria dysfunction leads to Caspase-1-induced pyroptosis. (**A**) Changes in ROS level in TNBC cells treated with indicated reagents, as detected via flow cytometry. (**B**,**C**) Western blots detected expression of Caspase-1 and GSDMD in TNBC cells treated with indicated reagents. (**D**) LDH released from TNBC cells treated in indicated reagents was assessed using LDH assay kits. Bar graphs are shown as mean ± SD of experimental triplicates (one-way ANOVA, *** *p* < 0.001). TNBC, triple-negative breast cancer; ROS, reactive oxygen species; Nig, nigericin; NAC, N-acetylcysteine; GSDMD, gasdermin-D; LDH, lactate dehydrogenase.

Previous studies showed that Caspase-1 activation was responsible for the GSDMD-dependent pyroptosis [11,12]. Mitochondria dysfunction, which was accompanied by increased levels of mitochondrial reactive oxygen species (mROS), which was caused by Caspase-1 activation [33,34]. Herein, we showed that nigericin induced pyroptosis via the Caspase-1/GSDMD pathway (Figure 1 and Figure 2) and mitochondria dysfunction in TNBC cells (Figure 3). We hypothesized that nigericin activated Caspase-1 by inducing mitochondria dysfunction and ROS accumulation in TNBC cells. Indeed, nigericin treatment increased the ROS level in TNBC cells (Figure 4A). Previous studies showed the mROS accumulation resulted in mitochondria membrane damage and increased permeabilization, allowing the release of mitochondrial DNA (mtDNA) from mitochondria, thus leading to decreased mtDNA being present in mitochondria [35,36]. We also found that the mtDNA decreased after nigericin treatment in TNBC cells (Figure S3A). In addition, treatment with N-acetylcysteine (NAC), which is an antioxidant commonly used for scavenging ROS and protecting mitochondria [37], prevented nigericin-induced ROS production (Figure 4A) and pyroptosis (Figure S3B). The nigericin-increased level of cleaved Caspase-1 was dampened in NAC pre-treated cell lines (Figure 4B). The increased level of N-terminal fragments of GSDMD upon nigericin treatment was also reversed (Figure 4B). Furthermore, neither cleavage of GSDMD (Figure 4C) nor elevated LDH release (Figure 4D) was observed in Caspase-1 knockdown TNBC cells upon nigericin treatment, which verified that nigericin induced pyroptosis in a Caspase-1-dependent manner. Together, these data suggested that the nigericin-induced pyroptosis in TNBC cells was elicited via mitochondria dysfunction and the following Caspase-1 activation.

### 3.5. Nigericin-Mediated Mitochondria Dysfunction Induces Caspase-3 Activation and Apoptosis

**Figure 5:**
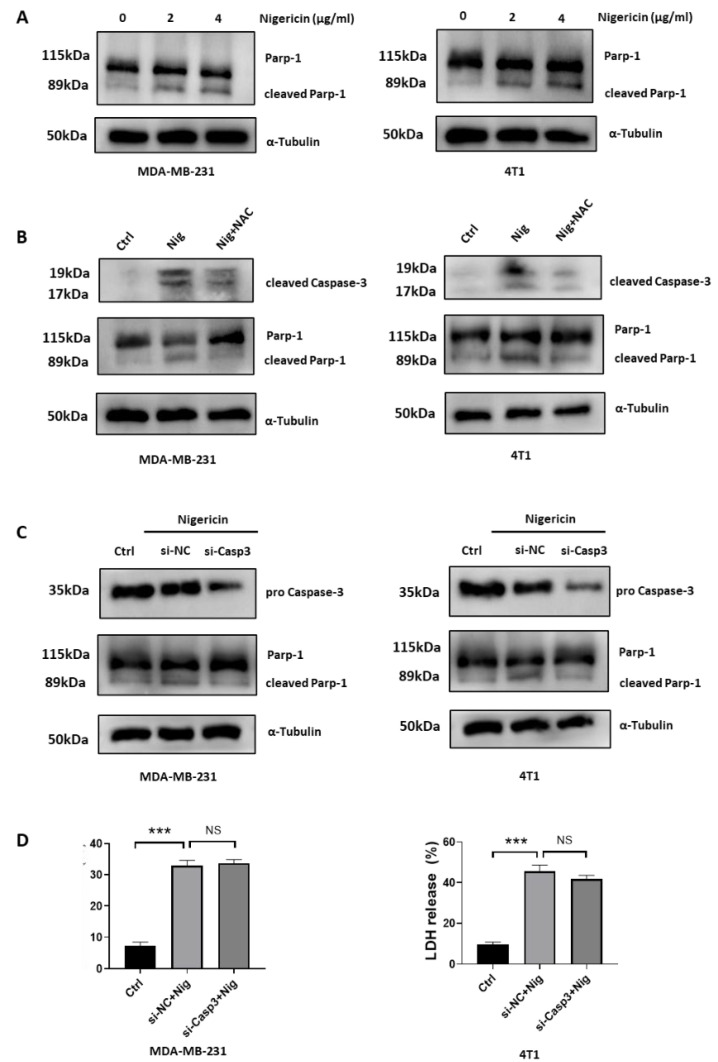
Nigericin-mediated mitochondria dysfunction leads to Caspase-3 dependent apoptosis. (**A**–**C**) Changes in Parp-1 and Caspase-3 in TNBC cells treated with indicated reagents, as analyzed via western blots. (**D**) LDH released from TNBC cells treated with indicated reagents was assessed using LDH assay kits. Each bar represent means± SD of experimental triplicates (one-way ANOVA, *** *p* < 0.001). TNBC, triple-negative breast cancer; NAC, N-acetylcysteine; Nig, nigericin; GSDMD, gasdermin-D; LDH, lactate dehydrogenase.

We showed that nigericin induced both pyroptosis and apoptosis in TNBC cells (Figure 1). Evidence showed that ROS accumulation mediated the alteration of mitochondria membrane permeabilization and caused the release of mitochondrial contents, such as cytochrome C, thus triggering the mitochondrial apoptosis [38,39,40]. Activated Caspase-3 cleaves Parp-1 to prevent DNA repair and promote apoptosis [41]. We observed increased levels of cleaved Parp-1 in nigericin-treated TNBC cells (Figure 5A). Next, we sought to test whether nigericin-mediated apoptosis was rescued by reducing the ROS accumulation in mitochondria. We showed that NAC could reverse nigericin-mediated ROS accumulation and protect mitochondria. Consistently, pre-treatment with NAC reversed the increased level of cleaved Parp-1 (Figure 5B), thus rescuing the nigericin-induced TNBC cells apoptosis (Figure S3B). The cytosol cytochrome C activates apoptosome to mediate Caspase-3 cleavage and trigger apoptosis [38,39,40]. Although activated Caspase-3 can cleave GSDME to induce pyroptosis and cleave Parp-1 for apoptosis, our above data suggested that Caspase-3/GSDME was not involved in nigericin-mediated pyroptosis (Figure 1). Thus, we speculated that the upregulated cleaved Caspase-3 participated in nigericin-mediated apoptosis. Consistently, the upregulated Caspase-3 cleaved via nigericin treatment could be restored via NAC pre-treatment (Figure 5B). In addition, the cleaved Parp-1 upon nigericin treatment decreased upon Caspase-3 knocking down (Figure 5C), suggesting the crucial role of Caspase-3 in nigericin-induced apoptosis. On the other hand, the release of LDH in nigericin-treated TNBC cells was not impacted upon Caspase-3 knocking down (Figure 5D), which further verified that Caspase-3 was not involved in nigericin-induced pyroptosis. Taken together, these data suggested that nigericin-mediated mitochondria dysfunction induced apoptotic cell death via Caspase-3 activation.

### 3.6. Nigericin Plus Anti-PD-1 Shows Synergistic Anti-Cancer Effect

**Figure 6:**
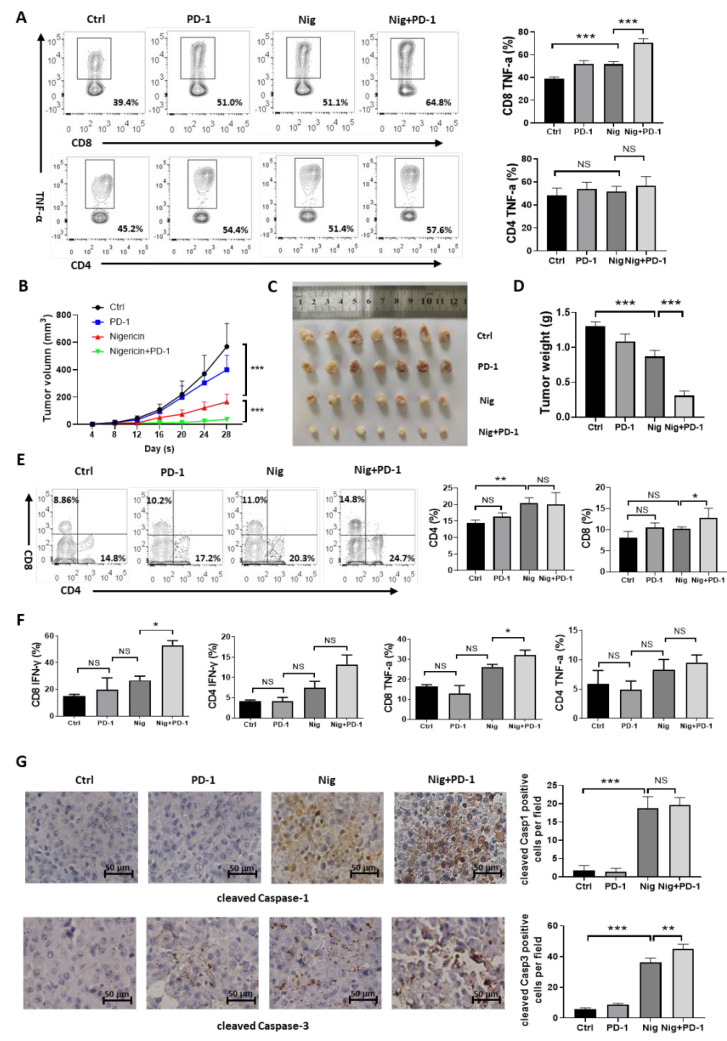
Nigericin combined with anti-PD-1 is an effective anti-tumor strategy. (**A**) Flow cytometry analysis of TNF-α secreted by CD4+ and CD8+ T cells from human PBMCs co-cultured with MDA-MB-231 and treated with indicated reagents. Each bar represents mean ± SD (one-way ANOVA, *** *p* < 0.001). (**B**) 4T1 cells were injected orthotopically into left inguinal mammary fat pad of BALB/c mice, which were treated with either nigericin (subcutaneous) or anti-PD-1 antibody (intraperitoneal) alone or together. Tumor volumes were monitored every 3–4 days. Displayed are means ± SD of different groups (two-way ANOVA, *** *p* < 0.001). (**C**,**D**) Representative image (**C**) and tumor weights (**D**) of 4T1 xenografts. Each bar represents mean ± SD (one-way ANOVA, *** *p* < 0.001). (**E**) Proportions of infiltrated CD4 and CD8 in tumors were detected via flow cytometry. Each bar represents mean ± SD (one-way ANOVA, * *p* < 0.05, ** *p* < 0.01). (**F**) Flow cytometry analysis of IFN-γ or TNF-α secreted by CD4+ and CD8+ T cells. Displayed are means ± SD of different groups (one-way ANOVA, * *p* < 0.001). (**G**) Expression of cleaved Caspase-1 or cleaved Caspase-3 of tumors from each group, as detected via IHC. Each bar represents mean ± SD (one-way ANOVA, ** *p* < 0.01, *** *p* < 0.001). PD-1, programmed death-1; Nig, nigericin; PBMCs, human peripheral blood mononuclear cells; TNF, tumor necrosis factor; IHC, immuno-histochemistry.

The degree of T cell infiltration and function is tightly linked to the response of anti-PD-1 in cancer treatment. As pyroptosis in tumor cells strongly boosted inflammation and enhanced anti-tumor immune responses [14], we proposed that nigericin would augment the anti-tumor effect of immune checkpoint inhibitors in TNBC. To address whether nigericin induced-pyroptosis enhanced the anti-tumor effect of T cells with the combination of PD-1 antibody, MDA-MB-231 cells were cultured with human peripheral blood mononuclear cells (PBMCs) isolated from human healthy donors (Figure S4A), followed by treatment with nigericin, anti-PD-1 or their combination. As activated CD4+ and CD8+ T cells would secret TNF-α to exert cytotoxic effects on tumor cells [42], we performed flow cytometry analysis to detect TNF-α. We then observed significantly higher TNF-α secretion of CD8+ T cells and slightly higher TNF-α secretion of CD4+ T cells in anti-PD-1 and nigericin treatment groups, while T cells in combination therapy group showed an even higher TNF-α secretion level (Figure 6A). These results supported the idea that nigericin enhanced the anti-tumor effect of T cells by inducing pyroptosis of tumor cells. Moreover, nigericin could further augment the PD-1 antibody-mediated anti-tumor effect of T cells.

Next, we evaluated the synergistic anti-tumor effect of nigericin with anti-PD-1 antibody in the 4T1 xenograft model, which included relatively cold tumors and was resistant to immune checkpoint inhibitor treatment due to a lack of T cell infiltration and activation [43,44]. The 4T1 cells were orthotopically transplanted into the mammary fat pad of BALB/c mice. Mice were randomly divided into four groups and treated with the control, nigericin (subcutaneous) or anti-PD-1 antibody (intraperitoneal) alone and together. As expected, PD-1 antibody alone did not show significant anti-tumor effect, whereas nigericin showed moderate anti-tumor effect. The combination of nigericin and anti-PD-1 antibody almost completely suppressed tumor growth (Figure 6B–D). Consistently, the tumor infiltrated CD4+ or CD8+ T cells were increased in the nigericin-treated and combination therapy groups (Figure 6E), suggesting that nigericin-mediated pyroptosis modulated the tumor microenvironment to facilitate the T cell infiltration, thus turning cold tumors into hot tumors. The levels of TNF-α and IFN-γ secreted by CD4+ or CD8+ cells were higher in the nigericin and anti-PD-1 combination therapy groups (Figure 6F and Figure S4C). In accordance with above results, cleaved Caspase-1 and cleaved Caspase-3 also increased in the nigericin-treated group (Figure 6F). In addition, we did not observe that nigericin impacted the expressions of PD-1 or PD-L1 in immune cells and cancer cells (Figure S4D). These data confirmed the synergistic anti-tumor effect of nigericin with anti-PD-1 antibody.

Meanwhile, the systematic side effects of these treatments were assessed in vivo. No noticeable histological toxicity was observed in the tissues from heart, liver, spleen, lung and kidney (Figure S5A). Hematological parameters, including white blood cells, hemoglobin, aspartate aminotransferase, alanine aminotransferase, albumin and creatinine, were in the normal range when treatment was completed (Figure S5B). In conclusion, these results suggested that applying nigericin was an effective strategy for sensitizing TNBCs to immune checkpoint blockage therapy with acceptable systematic side effects.

## 4. Discussion

Although numerous agents are being exploited to treat TNBC, effective therapeutic drugs are still very limited [2,3,4]. Benefits from immune checkpoint inhibitors were demonstrated in the Impassion130 and TONIC clinical trials [6,7]. In addition, the Impassion031 clinical trial revealed that anti-PD-1 could improve the response rate of chemotherapies in neoadjuvant treatment of TNBC [45]. However, the response rate of checkpoint inhibitors in TNBC remains relatively low. Treatments that modulate the tumor microenvironment to induce lymphocytes infiltration and enhance their anti-tumor capabilities are being actively developed to improve the therapeutic effect of checkpoint inhibitors. Clinical trials of Imbrave150 and Keynote426 showed that targeting blood vessels was an effective strategy to modulate the tumor microenvironment and improve the anti-cancer effect of immune checkpoint inhibitors in hepatic and renal cancers [46,47]. Although the anti-angiogenesis therapy showed synergistic effect with immune checkpoint inhibitors in TNBC, the therapeutic response remained unsatisfied [48]. Thus, there is a pressing need to develop effective ways to modulate the immune-microenvironment, as well as boost the therapeutic effect of checkpoints inhibitors in TNBC. Recently, pre-clinical studies showed combination therapy with agents inducing specific anti-tumor immune responses, such as cancer peptide-based vaccines or epitope–peptide vaccines, providing unique and effective options for active immunotherapy [49,50].

**Figure 7:**
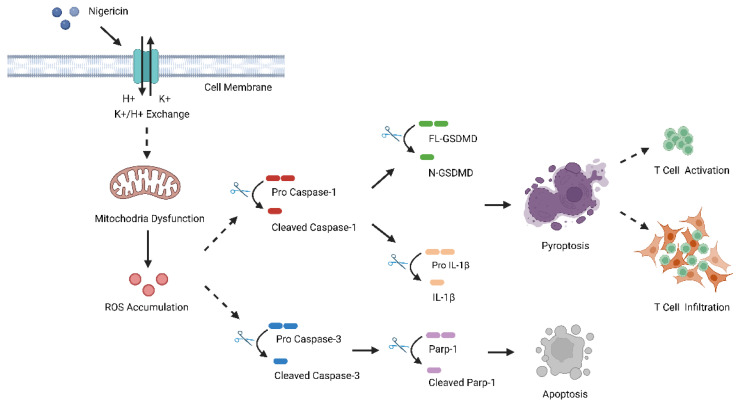
Schematic summary of mechanism of Nigericin in inducing anti-tumor immune response. Nigericin caused cellular potassium efflux and mitochondrial dysfunction, leading to mitochondrial ROS accumulation, as well as activation of Caspase-1/GSDMD-mediated pyroptosis and Caspase-3-dependent apoptosis in TNBC cells. Finally, nigericin-induced pyroptosis could amplify anti-tumor immune response by enhancing infiltration and anti-tumor effects of CD4+ and CD8+ T cells.

Nigericin, which is known as a K+/H+ ionophore, can perturb TCA cycle and oxidative phosphorylation by causing mitochondria dysfunction [30,31]. Although nigericin was proven to be a promising anti-tumor agent in multiple cancers [19,20,21,22], the anti-cancer mechanisms of nigericin were not fully understood. Here, we first demonstrated that nigericin inhibited TNBC cell viability by inducing concurrent apoptosis and pyroptosis. To be specific, nigericin induced ROS accumulation and mitochondria dysfunction of TNBC cells, thereby increasing the level of cleaved Caspase-3 and activating the apoptotic pathway. Meanwhile, the mitochondria dysfunction also resulted in the activation of Caspase-1/GSDMD cascades, leading to pyroptosis in TNBC cells (Figure 7). Thus, nigericin treatment inhibited the growth of TNBC in vitro (Figure S1A) and in vivo (Figure 6B–D).

Previous studies showed that pyroptosis amplified the anti-cancer immune response by turning “cold” tumors into “hot” tumors [14,15,16,51]. Nigericin-mediated pyroptosis is accompanied by the release of large amount of pro-inflammatory factors, such as IL-1β, and damage-associated molecular patterns (DAMPs), such as HMGB1, to induce strong immune responses [12,13,52]. In our study, when pre-treated with nigericin, TNBC cell-released IL-1β significantly boosted the TNF-α secretion of CD8+ T cells. When nigericin was combined with PD-1 antibody, T cells could secrete even higher TNF-α, suggesting the synergistic effect of nigericin with PD-1 on immune response of cancer cells (Figure 6A). Our in vivo experiment showed that nigericin treatment induced CD4+ and CD8+ T cells to infiltrate into 4T1 tumors, which was regarded as a cold tumor model [43,44]. These observations suggested that the inflammation triggered by pyroptosis upon nigericin treatment turned “cold” tumors into “hot” tumors. Consequently, anti-PD-1 treatment with nigericin almost completely inhibited 4T1 tumor growth. Moreover, the safety of nigericin was also verified, and no obvious systematic side effects were observed in vivo in our study, as nigericin is widely used in animal experiment and has satisfied safety regulators for decades [22,53,54]. The limitation of our study is that the animal experiments were conducted in the orthotopic model of 4T1 mouse breast cancer cells. Further studies using human breast cancer cells will be performed in the humanized mouse models. Moreover, whether the expression of Gasdermin family proteins, such as GSDMD, can be utilized as biomarkers to predict the immune status of tumors and effectiveness of immunotherapy and nigericin treatment may need to be further tested in the future.

## 5. Conclusions

Our study demonstrated the anti-tumor effect of nigericin on TNBCs by inducing concurrent Caspase-1/GSDMD-dependent pyroptosis and Caspase-3-dependent apoptosis. Moreover, nigericin-induced pyroptosis enhanced the anti-tumor immune response, and nigericin combined with anti-PD-1 antibody showed a synergistic therapeutic effect. Our study suggests that nigericin may be a potential anti-tumor agent, especially in combination with immune checkpoint inhibitors used for TNBC treatment.

## Data Availability

Data are available on reasonable request. All data relevant to the study are included in the article or uploaded as online Supplementary Materials.

## Supplementary Materials

The following supporting information can be downloaded at: https://www.mdpi.com/article/10.3390/cancers15123221/s1. Figure S1 shows the supplementary data for Figure 1; Figure S2 shows the supplementary data for Figure 2; Figure S3 shows the supplementary data for Figure 4 and Figure 5; Figures S4 and S5 show the supplementary data for Figure 6; Tables S1 and S2 show the supplementary data for the Section 2; Figures S6–S15 show the raw western blot results for triplicate experiments.
