# Supplementary material for: Nigericin Boosts Anti-Tumor Immune Response via Inducing Pyroptosis in Triple-Negative Breast Cancer
Source: Cancers (Basel). 2023 Jun 16;15(12):3221. doi: 10.3390/cancers15123221 (PMC10296105; doi:10.3390/cancers15123221)

Supplementary data

These supplementary data contain 15 supplementary figures and 2 supplementary tables.

Supplementary Figures

Figure S1, related to Figure 1.

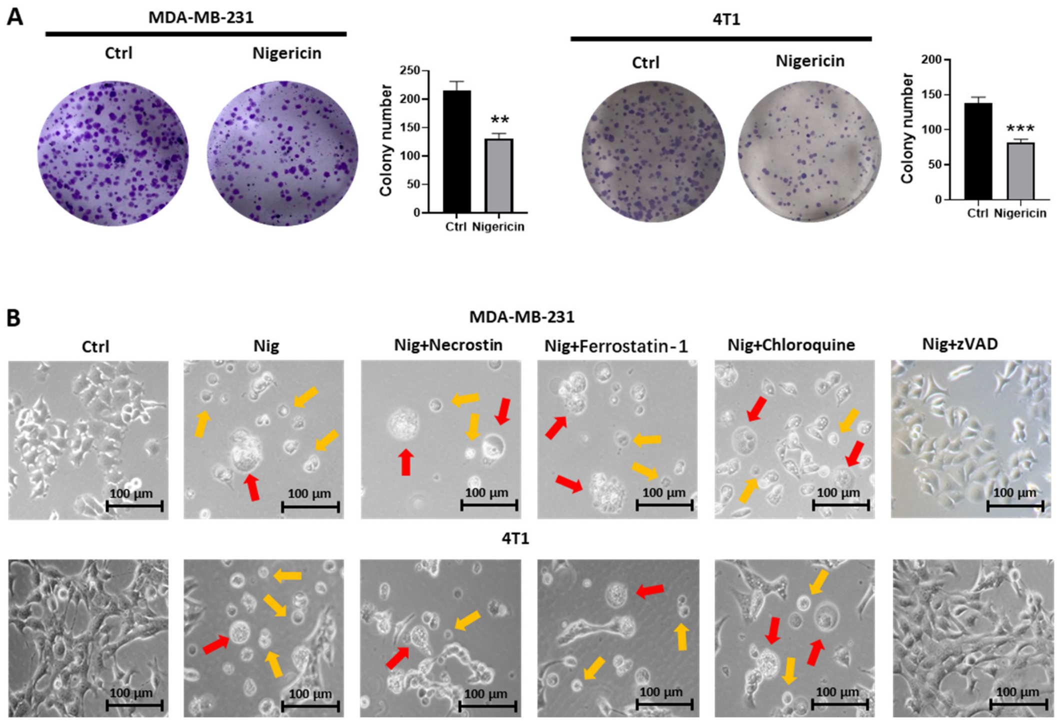

(A) Colony formation of MDA-MB-231 and 4T1 cells were inhibited by nigericin treatment (2 μg/ml). Each bar represents mean ± SD of experimental triplicates (T-test, ** $p < 0.01$, ***$p < 0.001$).

(B) Representative phase-contrast images of MDA-MB-231 and 4T1 cells. Cells were treated with nigericin (2 μg/ml) either alone or with indicated inhibitors, including necrostatin-1 (necroptosis inhibitor, 10 μM), ferrostatin-1 (ferroptosis inhibitor, 20 μM), chloroquine (autophagy inhibitor, 20 μM) and z-VAD (pan-Caspase inhibitor, 50 μM). Red arrows indicate pyroptotic cells, and yellow arrows indicate apoptotic cells.

Figure S2, related to Figure 2.

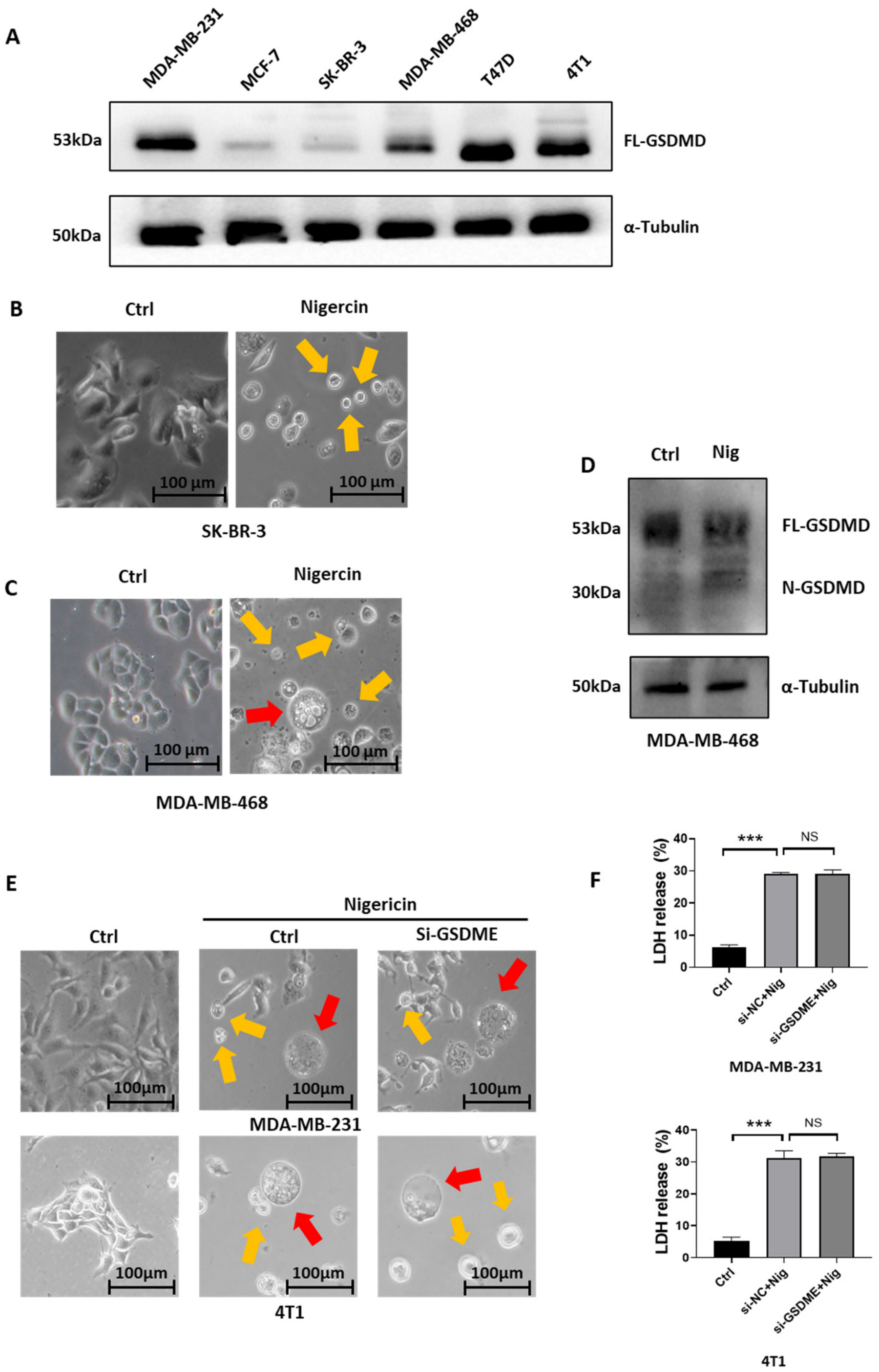

(A) Western blots detected the total GSDMD expressions of different breast cancer cell lines.

(B) Representative phase-contrast images of SK-BR-3 cells treated with nigericin or not. Red arrows indicate pyroptotic cells, and yellow arrows indicate apoptotic cells.

(C) Representative phase-contrast images of MDA-MB-468 cells treated with nigericin

or not. Red arrows indicate pyroptotic cells, and yellow arrows indicate apoptotic cells.

(D) Western blots detected the GSDMD changes in MDA-MB-468 cells upon nigericin treatment.

(E) Representative phase-contrast images of TNBC cells with GSDME knocking down or not, followed by nigericin treatment (2 μg/ml for 24 h). Red arrows indicate pyroptotic cells, and yellow arrows indicate apoptotic cells.

(F) LDH released from TNBC cells with GSDME knocking down or not, treated with nigericin (2 μg/ml for 12 h). Bar graphs represent means ± SD of experimental triplicates (one-way ANOVA, ***p* < 0.01).

Figure S3, related to Figure 4-5.

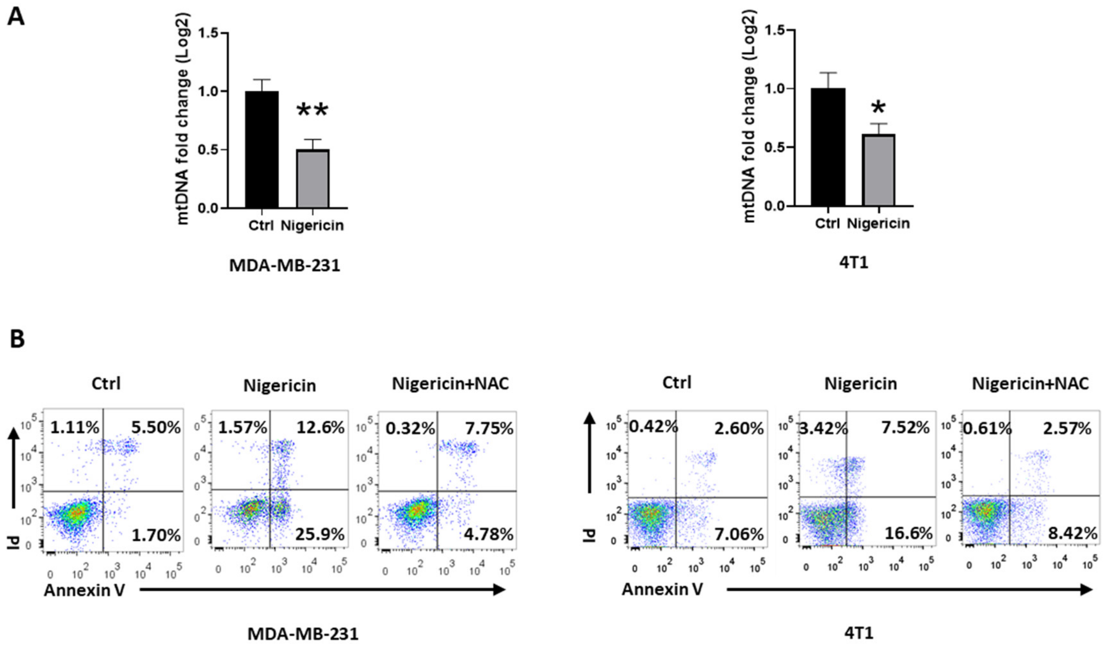

(A) Quantitative real-time PCR analysis of cytosolic mtDNA level in MDA-MB-231 and 4T1 cells upon nigericin treatment (2 μg/ml). Each bar represent mean ± SD of experimental triplicates (T-test, **p* < 0.05, ** *p* < 0.01).

(B) Flow cytometry detected PI and Annexin V stained TNBC cells treated with indicated reagents (nigericin 2 μg/ml, NAC 5 mM).

Figure S4, related to Figure 6.

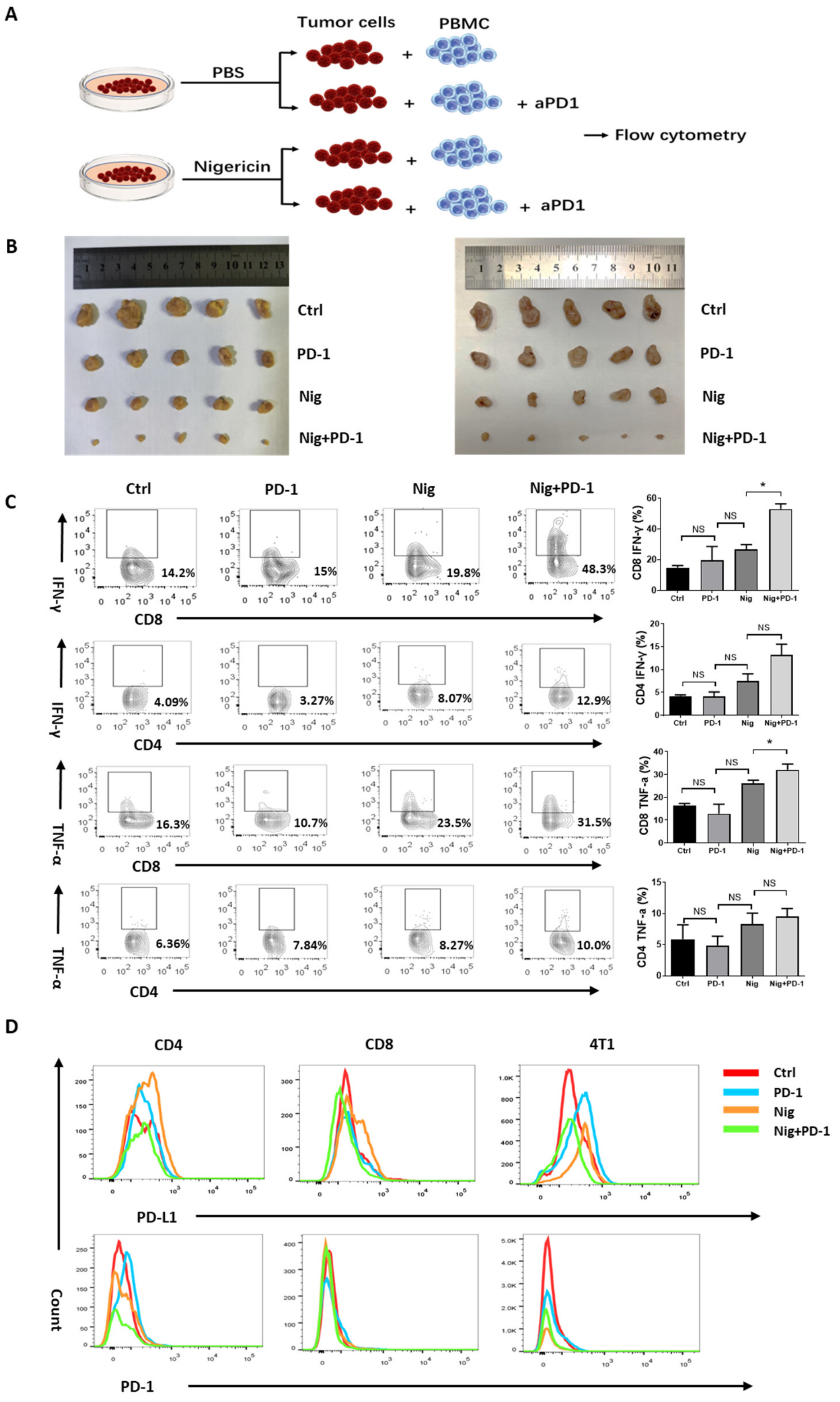

(A) Schematic diagram showed that MDA-MB-231 cells treated with either nigericin or not were cultured with PBMC isolated from human healthy donors, followed with or without anti-PD-1 treatment.

(B) Represent images of the other two replicate *in vivo* experiments.

(C) Flow cytometry analysis of IFN-γ or TNF-α secreted by CD4+ and CD8+ T cells. Displayed are means ± SD of different groups (one-way ANOVA, $*p < 0.05$).

(D) The expressions of PD-1 or PD-L1 in T cells and cancer cells, detected by flow cytometry.

Figure S5, related to Figure 6.

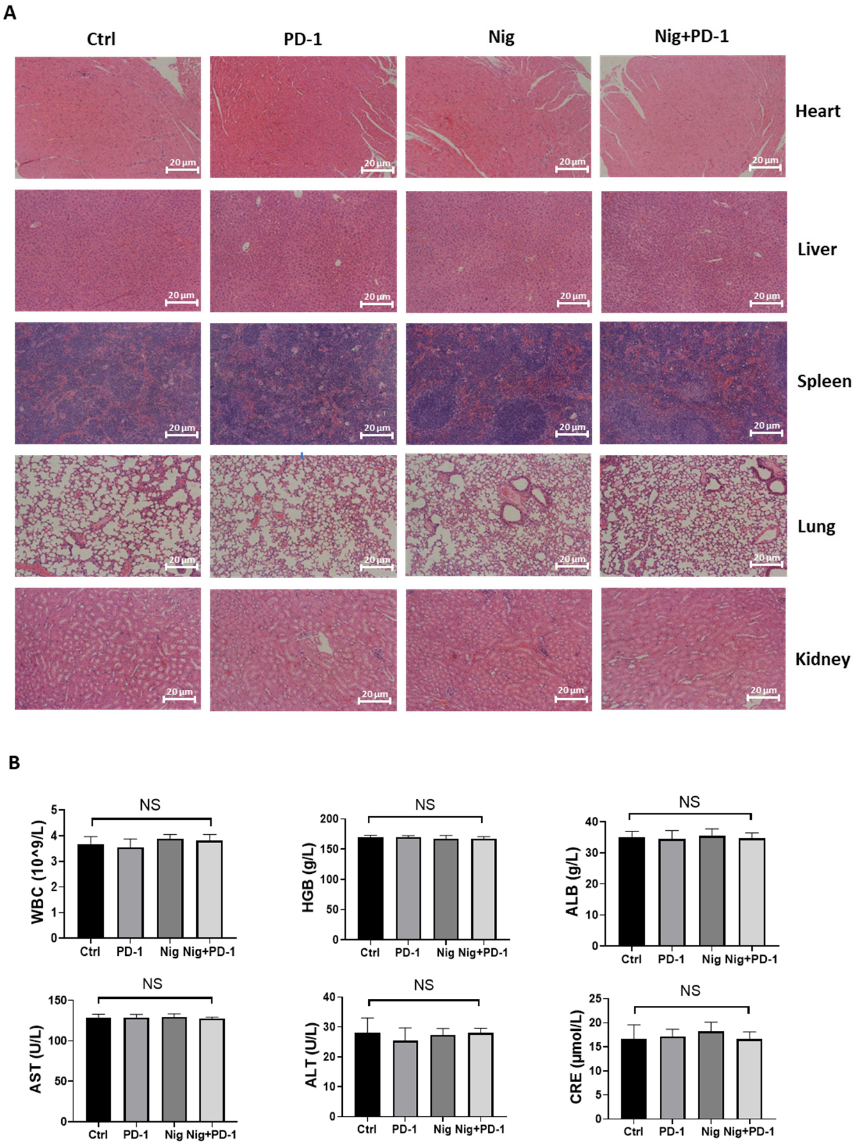

(A) Representative HE stained section images of mouse organs treated with indicated reagents, including the heart, lung, liver, kidney and spleen.

(B) Hematologic and biochemical values of mice treated with indicated reagents. Each bar represent mean ± SD (one-way ANOVA).

Table S1. Antibodies Used in WB, IF/IHC and FCM

Antibody	Company	Cat.	Application	Dilution rate
Mouse pro Caspase-1	Cell Signaling Technology	24232	WB	1:1000
Human pro Caspase-1	Cell Signaling Technology	3866	WB	1:1000
Mouse cleaved Caspase-1	Cell Signaling Technology	89332	WB	1:1000
Human cleaved Caspase-1	Cell Signaling Technology	4199	WB	1:1000
Pro Caspase-3	Cell Signaling Technology	9662	WB	1:1000
Cleaved Caspase-3	Cell Signaling Technology	9664	WB	1:1000
GSDMD	Cell Signaling Technology	93709	WB	1:1000
GSDME	Abcam	ab215191	WB	1:1000
PD-L1	Proteintech	17952-1-AP	WB	1:500
HMGB1	Proteintech	10829-1-AP	WB	1:2000
Caspase-1	Proteintech	22915-1-AP	WB	1:2000
α-Tubulin	Proteintech	66031-1-Ig	WB	1:5000
Parp-1	Proteintech	13371-1-AP	WB	1:2000
HRP-conjugated Affinipure Goat Anti-Rabbit IgG	Proteintech	SA00001-2	WB	1:3000
HRP-conjugated Affinipure Goat Anti-Mouse IgG	Proteintech	SA00001-1	WB	1:3000
GSDMD N-terminal	Affinity	AF4012	IF	1:100
Alexa Fluor® 594-conjugated Anti-Rabbit IgG	Cell Signaling Technology	8889S	IF	1:1000
Cleaved Caspase-1	Thermo Fisher	PA5-99390	IHC	1:100
Cleaved Caspase-3	Cell Signaling Technology	9579	IHC	1:250
APC human CD45	Biolegend	304012	FCM	5μl/10^6 cells
FITC human CD4	Biolegend	357406	FCM	5μl/10^6 cells
PerCP human CD8	Biolegend	344707	FCM	5μl/10^6 cells
PE human TNF-α	Biolegend	502908	FCM	5μl/10^6 cells
APC mouse CD45	Biolegend	103112	FCM	5μl/10^6 cells

FITC mouse CD4	Biolegend	100510	FCM	0.25μg/10^6 cells
PerCP mouse CD8	Biolegend	100731	FCM	0.25μg/10^6 cells
PE mouse TNF-α	eBioscience	12-7321-81	FCM	5μl/10^6 cells
APC mouse INF-γ	Biolegend	505809	FCM	1μg/10^6 cells
PE mouse PD-1	Biolegend	135205	FCM	1μg/10^6 cells
PE mouse PD-L1	Biolegend	124308	FCM	0.25μg/10^6 cells

WB, western blot; IF, immunofluorescence; IHC, Immunohistochemistry; FCM, flow cytometry;

Table S2. siRNA sequences

**Gene**	**siRNA Sequence**
Mouse Caspase-1	5′-GGGACCUAUGUGAUCAUGUCUCUAA-3′
Human Caspase-1	5′-GGAAGUGAAGAGAUCCUUCUGUAAA-3′
Mouse Caspase-3	5’-GCACTGGAATGTCAGCTCG-3’
Human Caspase-3	5’-GGAAUAUCCCUGGACAACA-3’
Mouse GSDMD	5’-CCGAGGUGCUGCAGACAAA -3’
Human GSDMD	5’-GUGUCAACCUGUCUAUCAA-3’
Mouse GSDME	5′-GCTGCAAACTCCATGTTAT-3′
Human GSDME	5’-CCAUUGCCUACGGUGUCAUTT-3’

Figure S6 related to Figure 1E

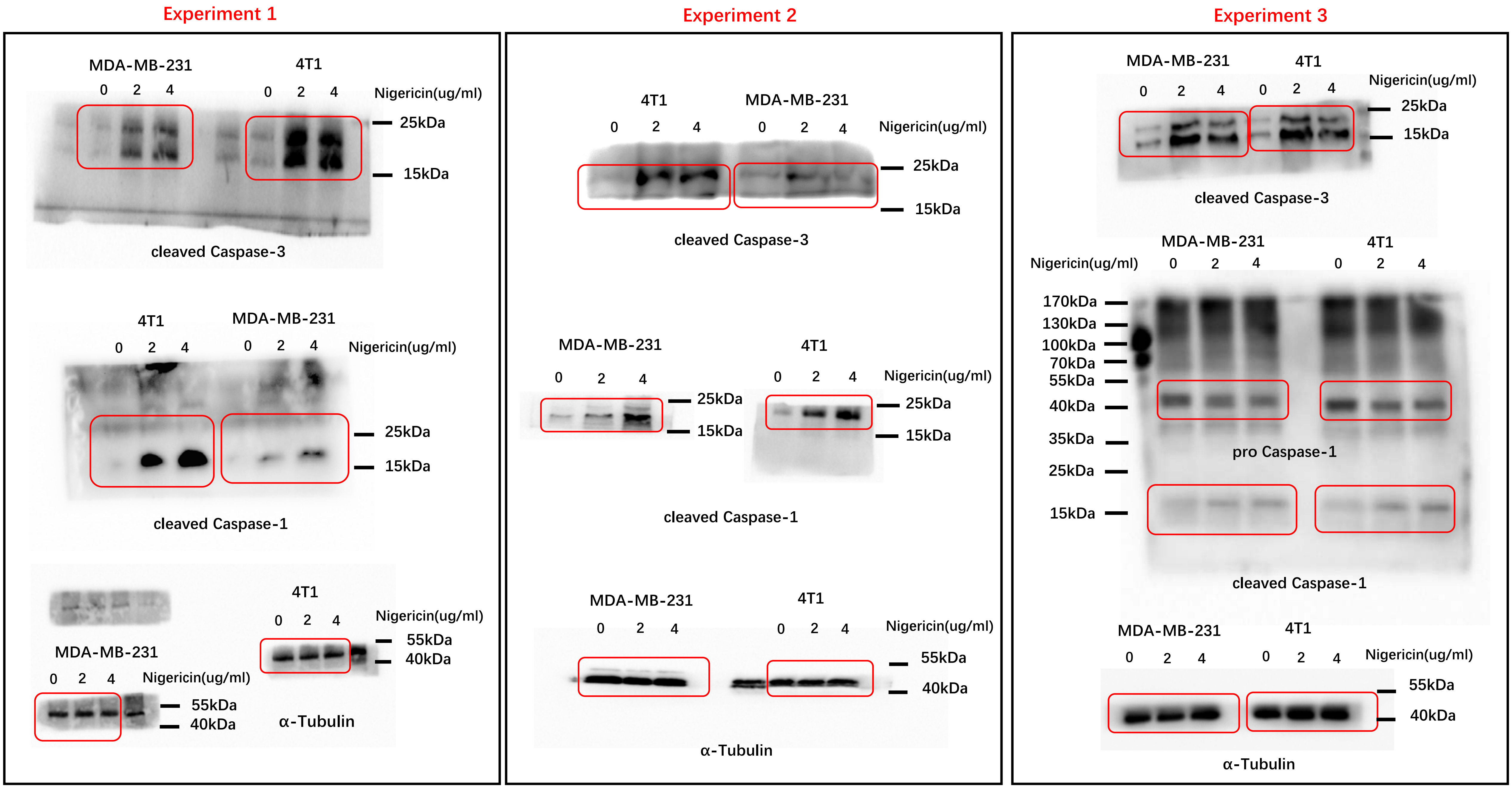

Figure S7 related to Figure 2A

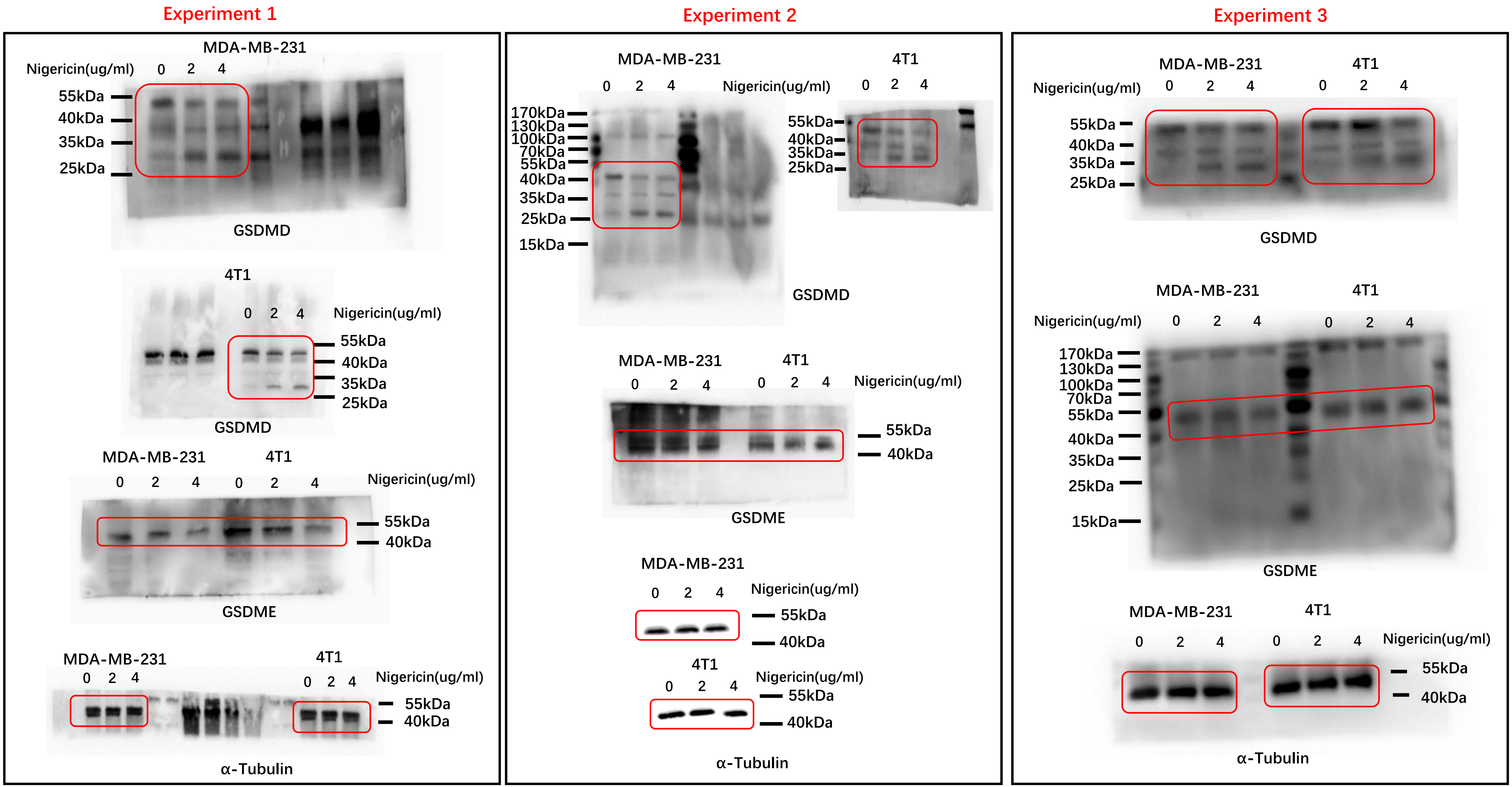

Figure S8 related to Figure 2E

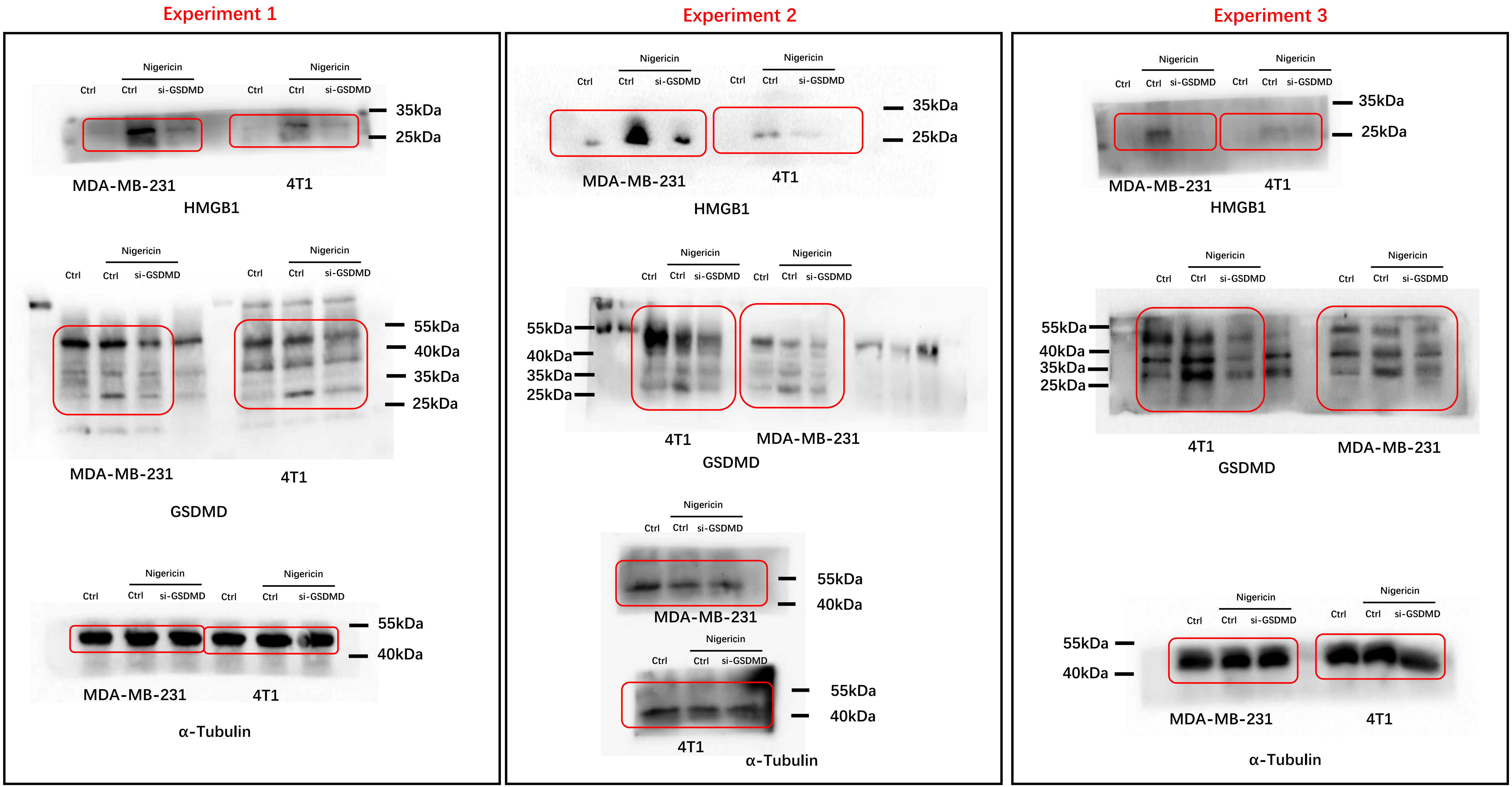

Figure S9 related to Figure 4B

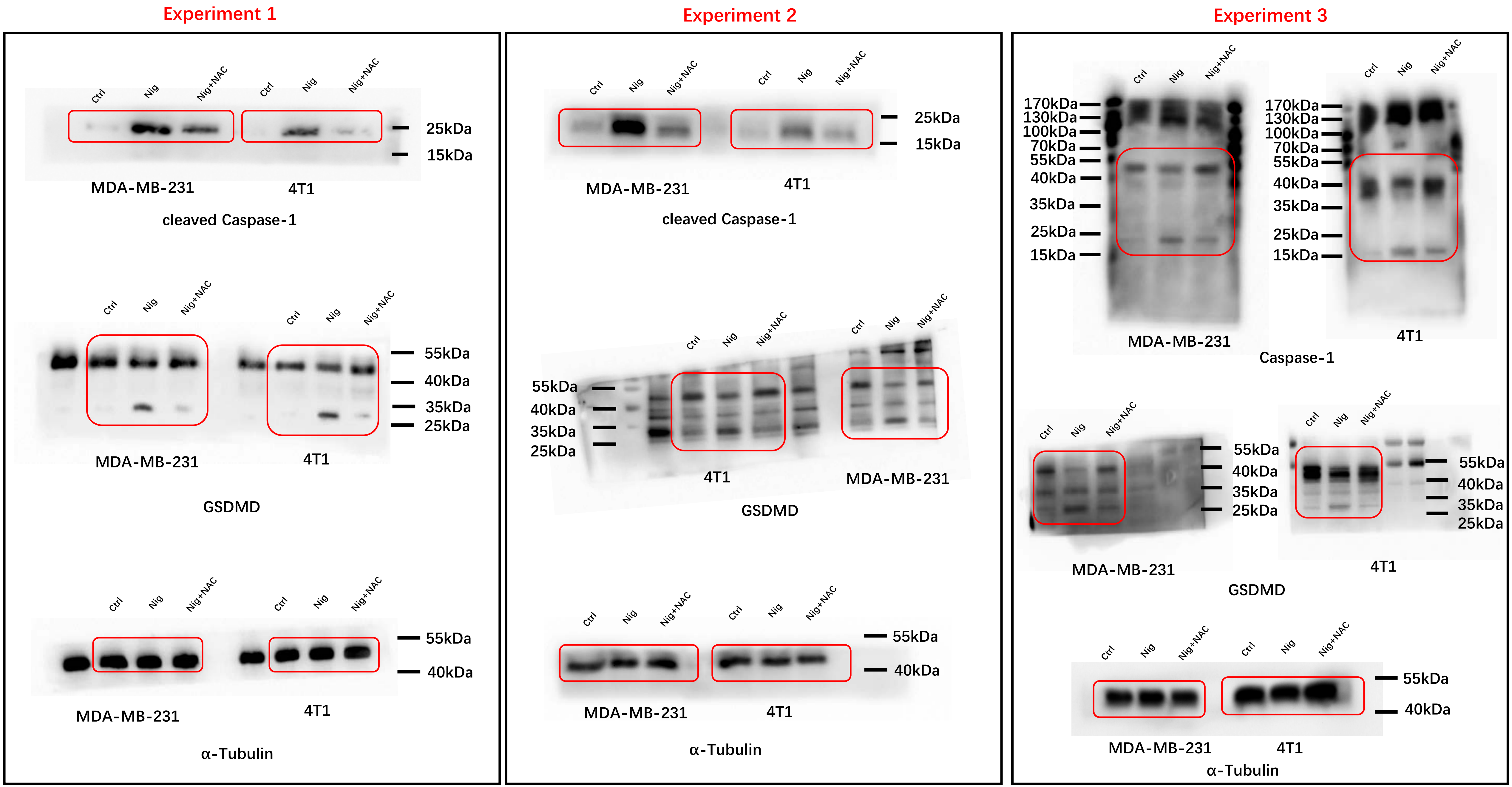

Figure S10 related to Figure 4C

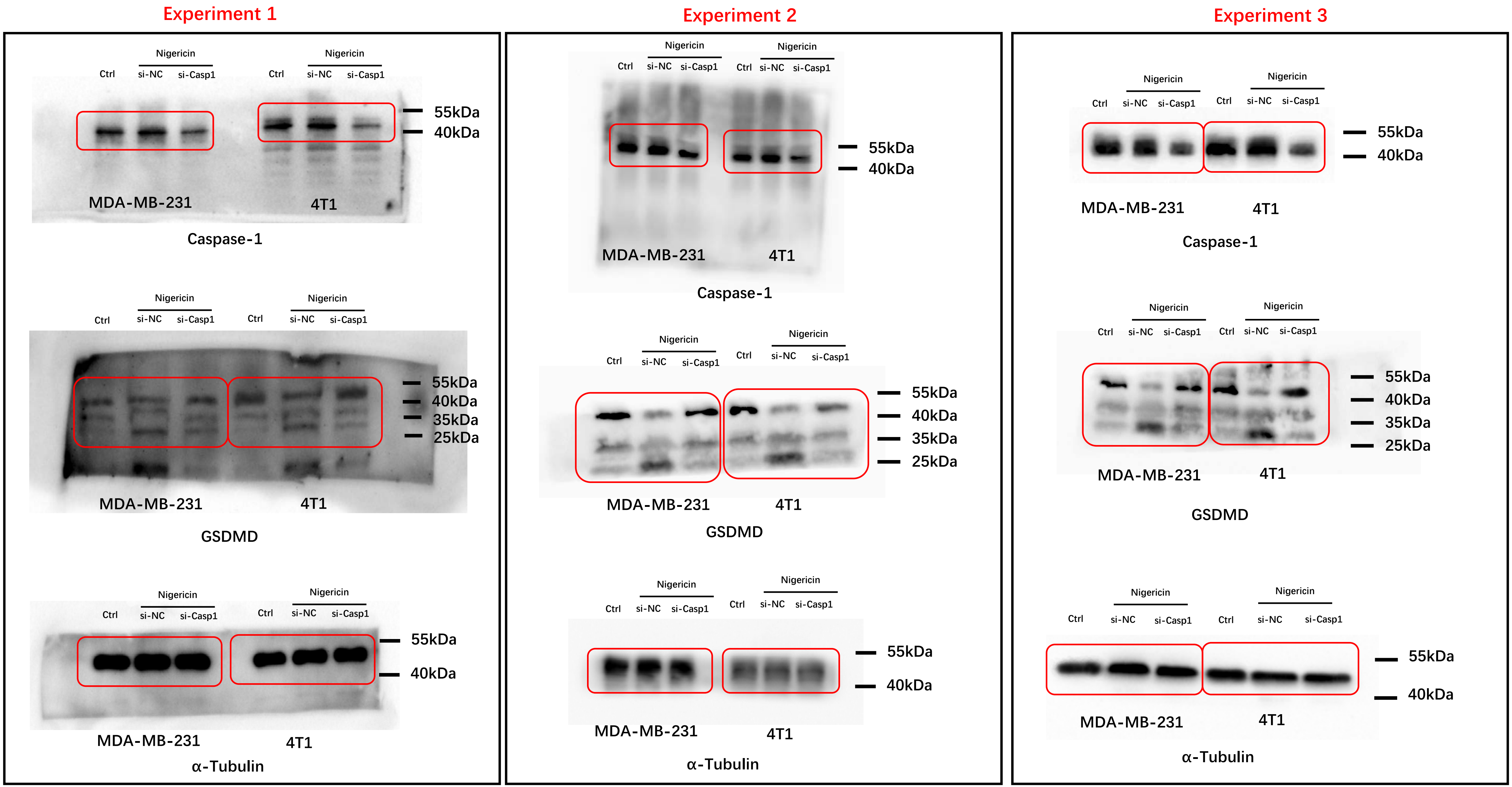

Figure S11 related to Figure 5A

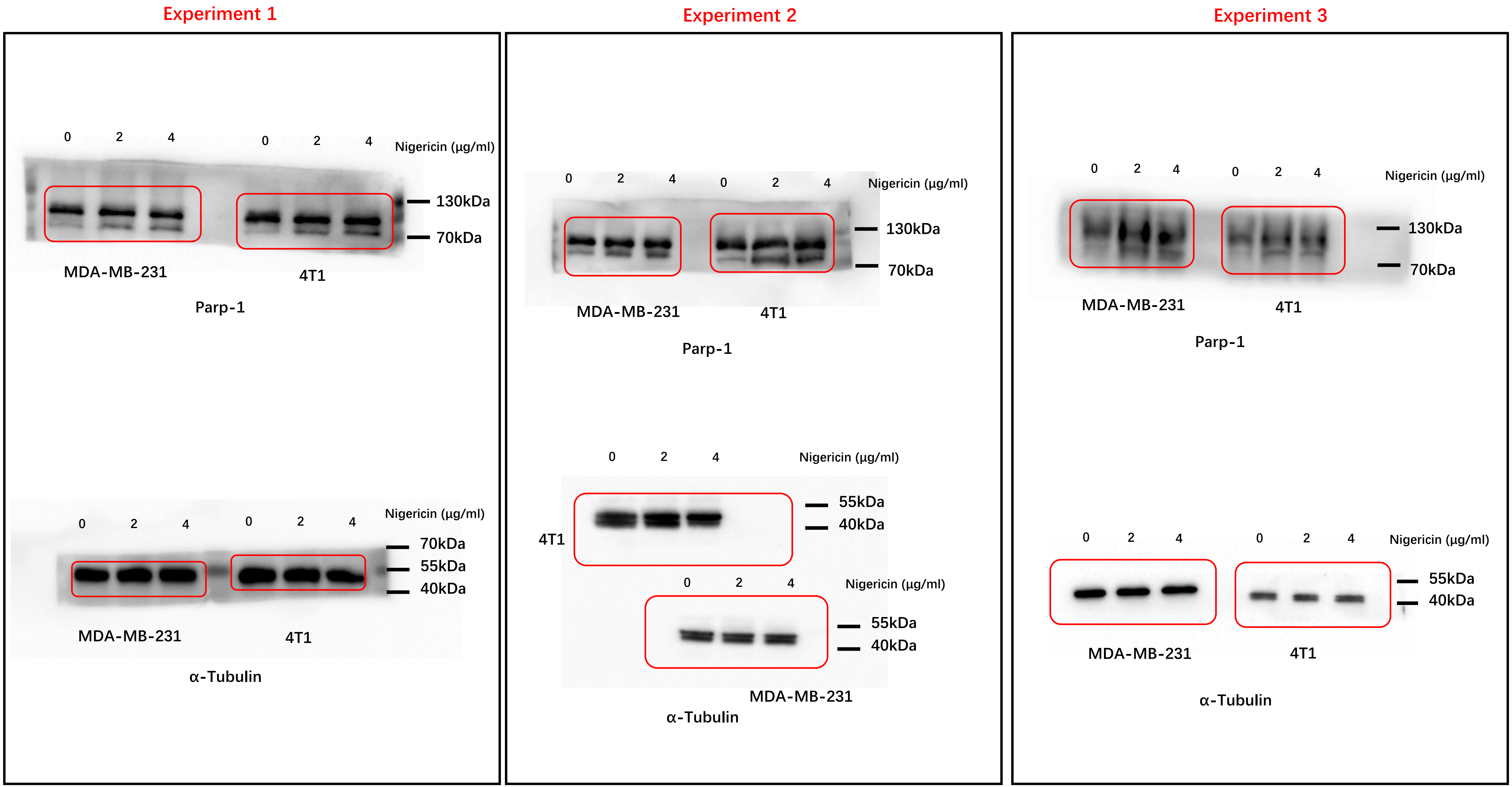

Figure S12 related to Figure 5B

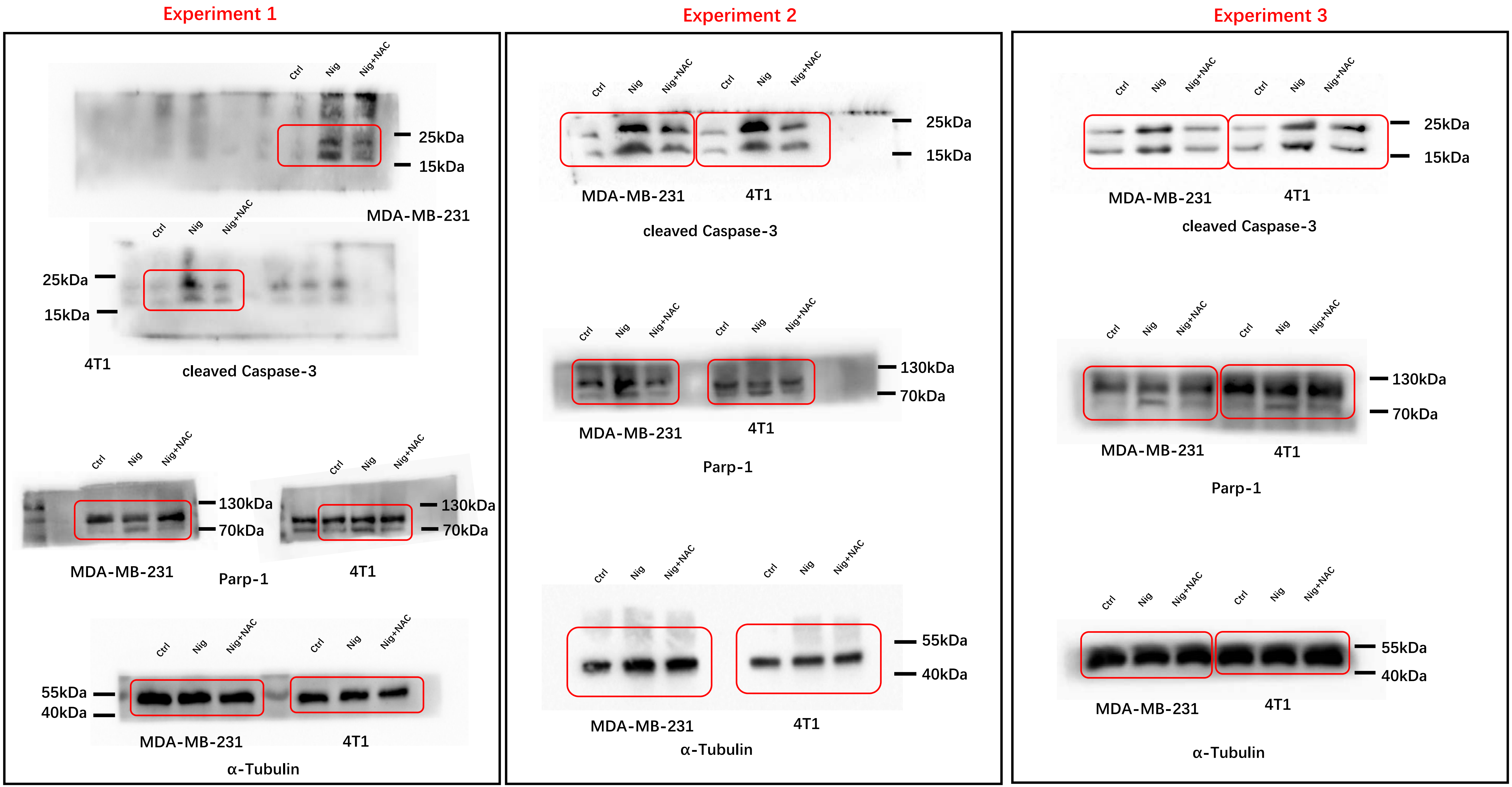

Figure S13 related to Figure 5C

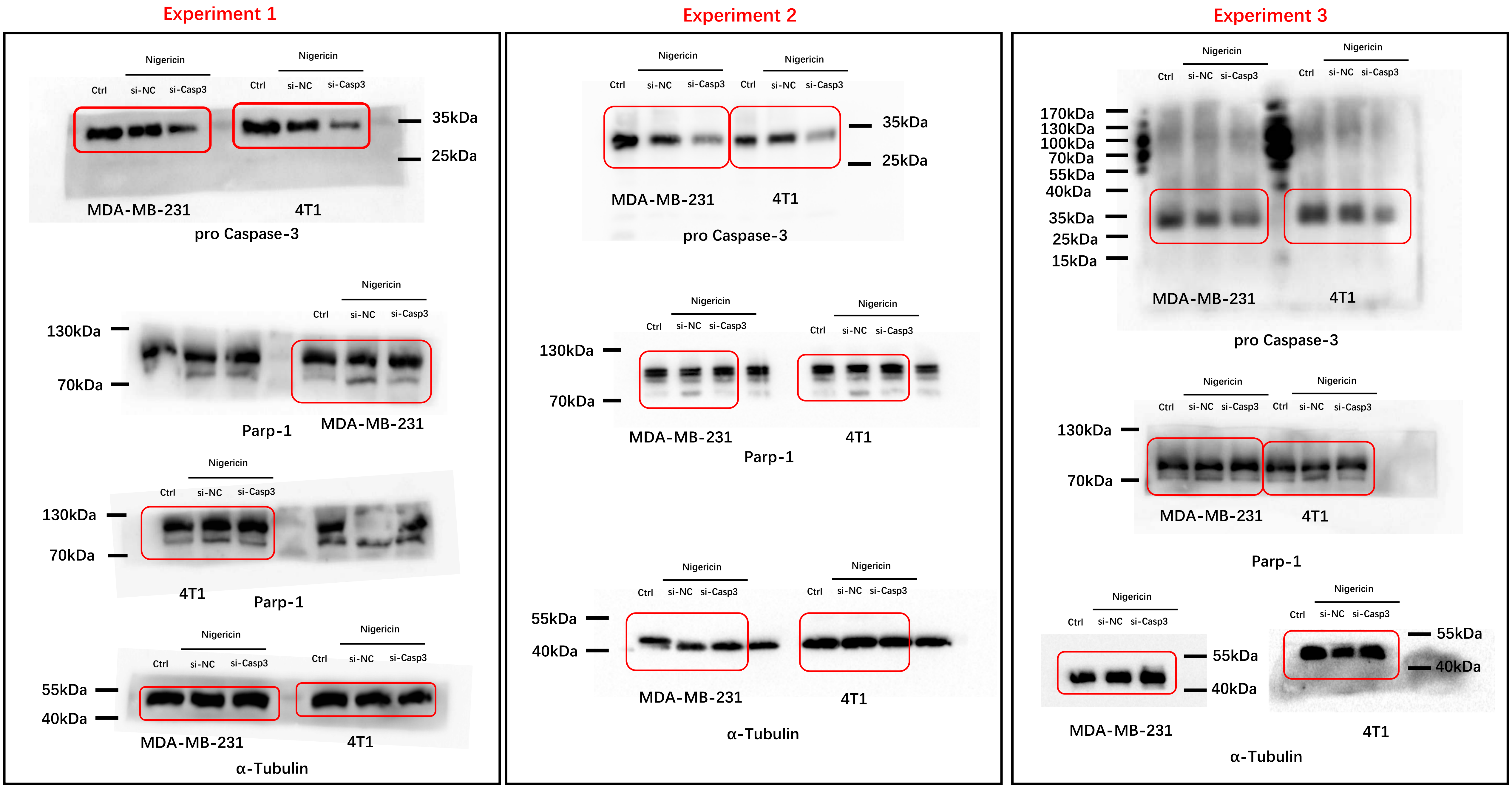

Figure S14 related to Figure S2A

Experiment 1

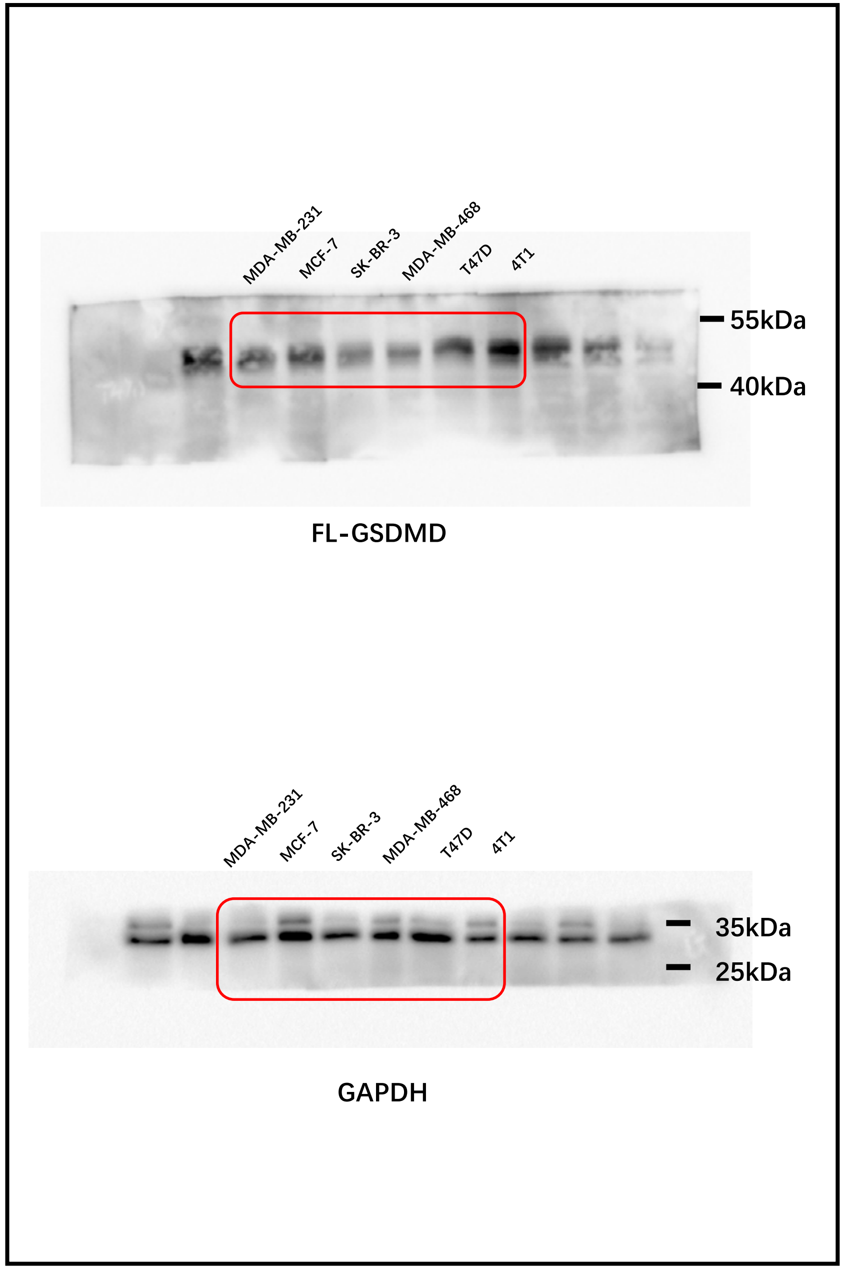

Experiment 2

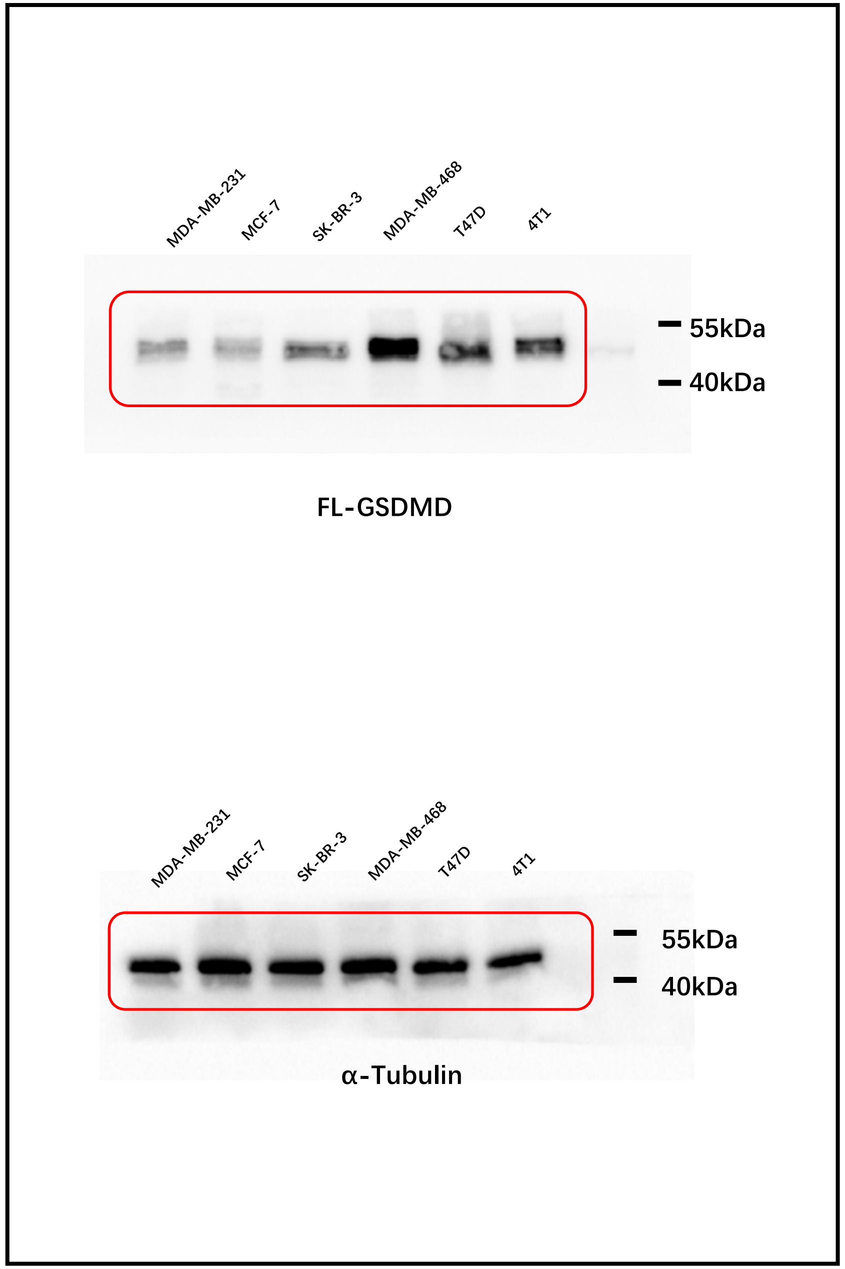

Experiment 3

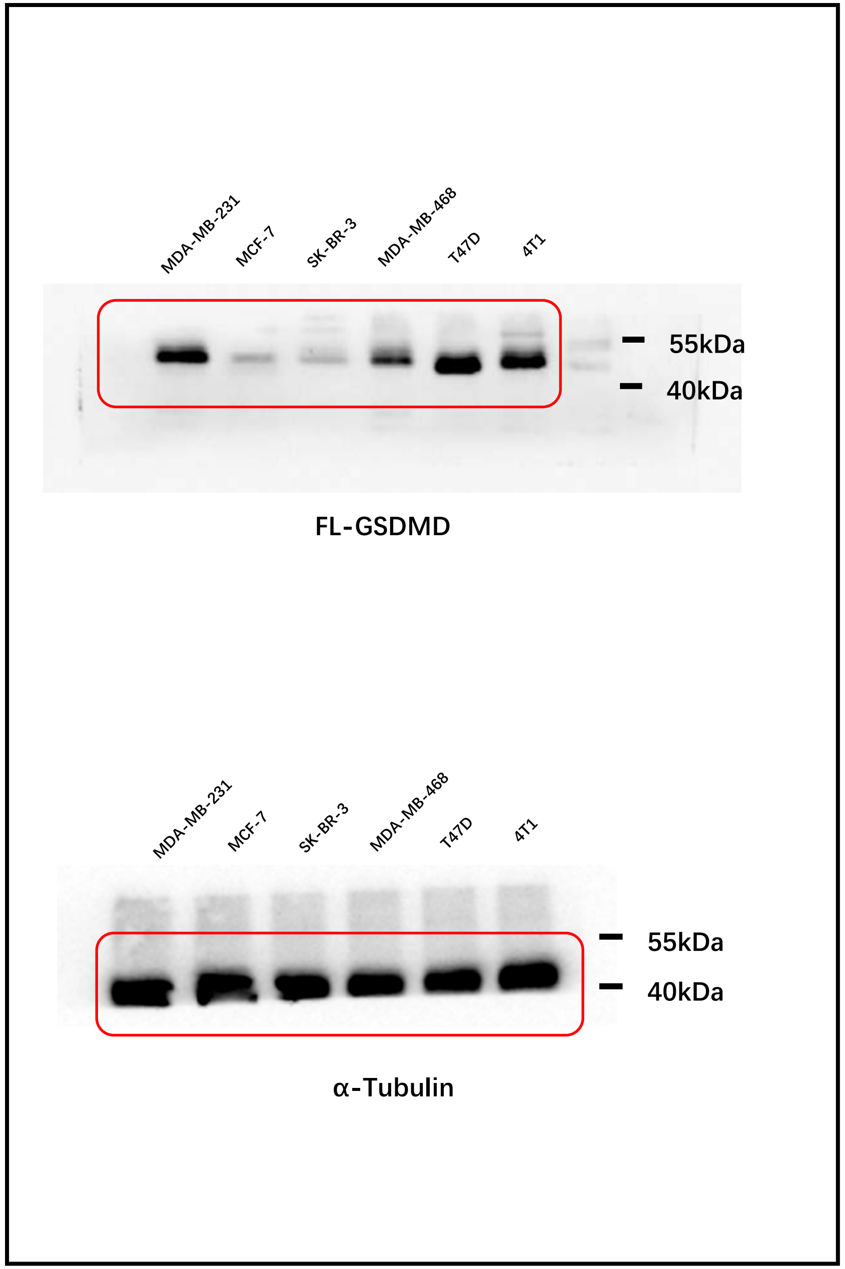

Figure S15 related to Figure S2D

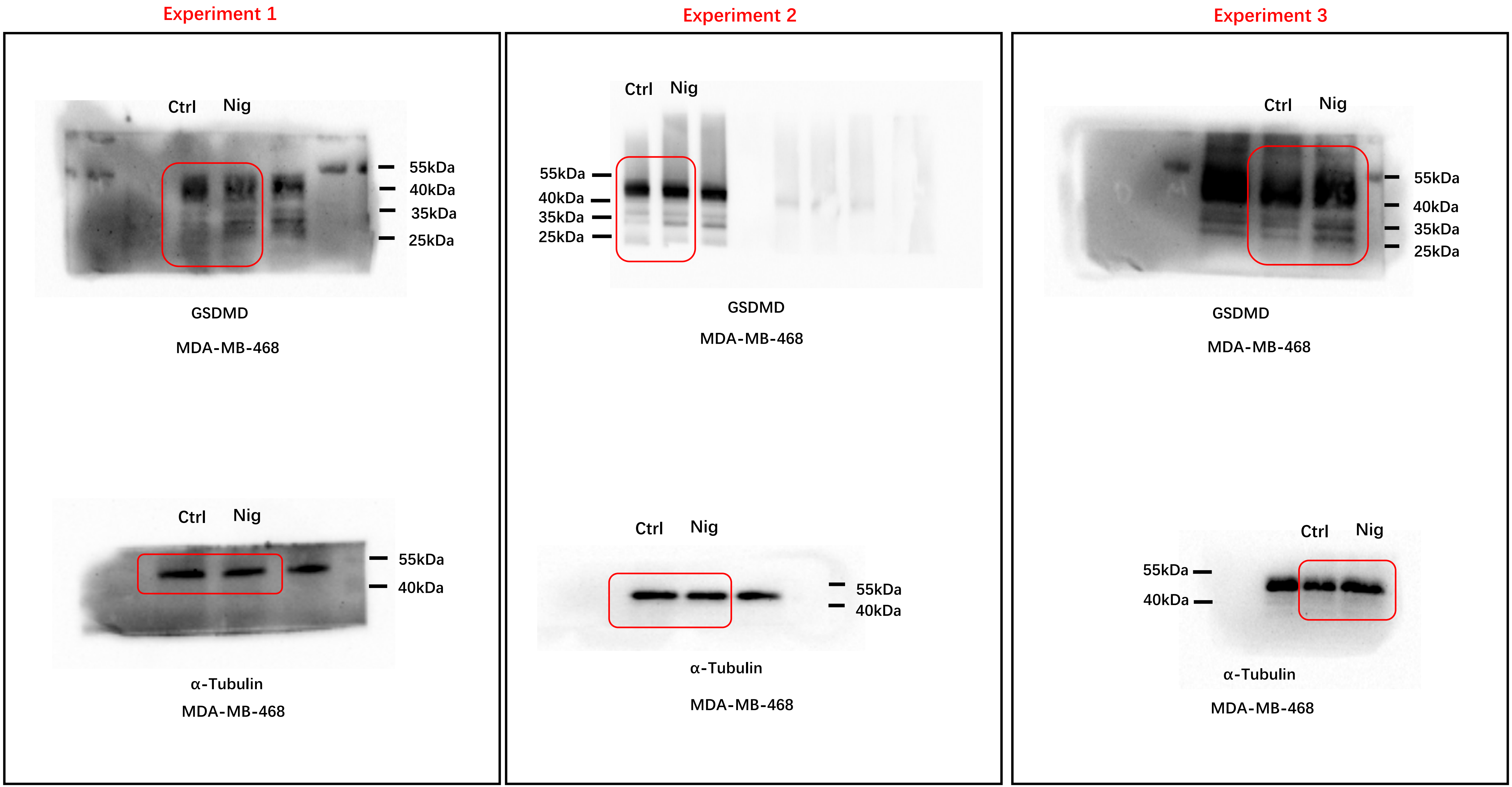

Supplement: Supplementary file 1 [file cancers-15-03221-s001.zip › cancers-2403722-supplementary.pdf]
